# Matrisome Properties of Scaffolds Direct Fibroblasts in Idiopathic Pulmonary Fibrosis

**DOI:** 10.3390/ijms20164013

**Published:** 2019-08-17

**Authors:** Linda Elowsson Rendin, Anna Löfdahl, Emma Åhrman, Catharina Müller, Thomas Notermans, Barbora Michaliková, Oskar Rosmark, Xiao-Hong Zhou, Göran Dellgren, Martin Silverborn, Leif Bjermer, Anders Malmström, Anna-Karin Larsson-Callerfelt, Hanna Isaksson, Johan Malmström, Gunilla Westergren-Thorsson

**Affiliations:** 1 Lung Biology, Department of Experimental Medical Sciences, Lund University, BMC C12, Lund 221 84, Sweden; 2 Division of Infection Medicine Proteomics, Department Clinical Sciences, Lund University, Lund 221 84, Sweden; 3 Department of Biomedical engineering, Lund University, Lund 221 84, Sweden; 4 Bioscience Department, Respiratory, Inflammation and Autoimmunity, IMED Biotech Unit, AstraZeneca, Mölndal 431 53, Sweden; 5 Department of Cardiothoracic Surgery and Transplant Institute, Sahlgrenska University Hospital, Gothenburg 413 45, Sweden; 6 Department of Respiratory Medicine and Allergology, Skåne University Hospital, Lund University, Lund 221 85, Sweden

**Keywords:** scaffold, decellularization, lung fibroblast, proteomics, extracellular matrix

## Abstract

In idiopathic pulmonary fibrosis (IPF) structural properties of the extracellular matrix (ECM) are altered and influence cellular responses through cell-matrix interactions. Scaffolds (decellularized tissue) derived from subpleural healthy and IPF lungs were examined regarding biomechanical properties and ECM composition of proteins (the matrisome). Scaffolds were repopulated with healthy fibroblasts cultured under static stretch with heavy isotope amino acids (SILAC), to examine newly synthesized proteins over time. IPF scaffolds were characterized by increased tissue density, stiffness, ultimate force, and differential expressions of matrisome proteins compared to healthy scaffolds. Collagens, proteoglycans, and ECM glycoproteins were increased in IPF scaffolds, however while specific basement membrane (BM) proteins such as laminins and collagen IV were decreased, nidogen-2 was also increased. Findings were confirmed with histology, clearly showing a disorganized BM. Fibroblasts produced scaffold-specific proteins mimicking preexisting scaffold composition, where 11 out of 20 BM proteins were differentially expressed, along with increased periostin and proteoglycans production. We demonstrate how matrisome changes affect fibroblast activity using novel approaches to study temporal differences, where IPF scaffolds support a disorganized BM and upregulation of disease-associated proteins. These matrix-directed cellular responses emphasize the IPF matrisome and specifically the BM components as important factors for disease progression.

## 1. Introduction

In idiopathic pulmonary fibrosis (IPF) the biomechanics and composition of the extracellular matrix (ECM) are altered causing a pathological phenotype associated with increased tissue stiffness and disorganized structures of the lung [1]. Today there is no real effective treatment for IPF with poor long-term survival, although there are treatments that may slow progression of IPF somewhat. Lung transplantation remains the most valid option for some patients, however, not all patients can be offered this treatment due to recipient co-morbidities or donor shortage. Although largely unknown, a combination of factors is believed to play a role in IPF, including ageing, epigenetic modifications, dysfunctional alveolar epithelium, along with persistent activation of lung fibroblasts that contributes to an increased accumulation of ECM with subsequent detrimental remodeling resulting in the loss of lung function and eventually end-stage lung disease [2,3,4]. Local signals from the ECM, e.g., stiffness and bound growth factors and cytokines, have been shown to influence cellular behavior such as migration, differentiation and proliferation, activities that are altered due to changes in the local microenvironment [5,6,7]. In a fibrotic lung, there is an imbalance in the turnover of ECM proteins causing excessive production and deposition of ECM proteins, forming a disease-specific organization and composition of the matrix [8,9,10]. The pathological mechanism underlying the initiation and progression of IPF is not fully understood, and there are no effective treatment options, highlighting the need to identify effective molecular targets for therapeutic interventions [11]. Lung tissue slices, decellularized for cellular removal, can serve as human structural matrices to study the important and complex interaction between cells and matrix [12,13]. In comparison to other cell culture systems, decellularized tissue (scaffolds) comprise a unique ex vivo system that more closely mimics the original intricate 3D milieu of the lung. Through this ex vivo model a better understanding of unknown key cellular mechanisms can be obtained in order to understand which ECM properties drive the formation of fibrotic tissue and which role the ECM of IPF scaffolds has in disease progression. The matrisome protein classification system defined by Naba et al., clearly describes the ECM components, subgrouping ECM matrisome core proteins (collagens, glycoproteins and proteoglycans) and ECM associated proteins such as ECM-affiliated proteins, ECM regulators, and secreted factors [14]. Distal lung tissue is mainly composed of fibrillar collagens I, III, V, and VI and the basement membrane (BM) collagen type IV [7]. Intertwined with collagen type IV are nidogens, perlecan, and laminins, which comprise the BM network, a protein complex facilitating epithelial and endothelial cell attachment and regulating cellular behavior [15]. Alterations of the BM structure and other ECM components affect both morphology and biomechanical properties of the tissues, identifying matrix stiffness as an important biomechanical signal for cell responses [16]. Tissue stiffening of the lung, caused by increased ECM deposition in the alveoli that leads to a loss in tissue elasticity, induces differentiation of fibroblasts into myofibroblasts, a response that in part could be de-activated when changing culture conditions in vitro from stiff to softer substrates [17]. In IPF, fibroblasts demonstrate an increased cellular stiffness, perhaps functioning as a positive feedback loop contributing to the formation of a non-compliant stiff lung tissue [18].

In this study we focused on the distal parenchymal matrisome properties of lung scaffolds derived from healthy donors and IPF patients in a unique 3D-ex vivo setting, mimicking pulmonary physiological conditions. Our hypothesis was that the matrisome properties, i.e., biomechanical properties and matrisome composition, of the ECM have a fundamental impact on cellular responses and may act as a mechanism in disease progression.

## 2. Results

### 2.1. Morphology and Biomechanical Properties of Native Tissue and Scaffolds Derived from Healthy and IPF Lung Tissue

**Figure 1:**
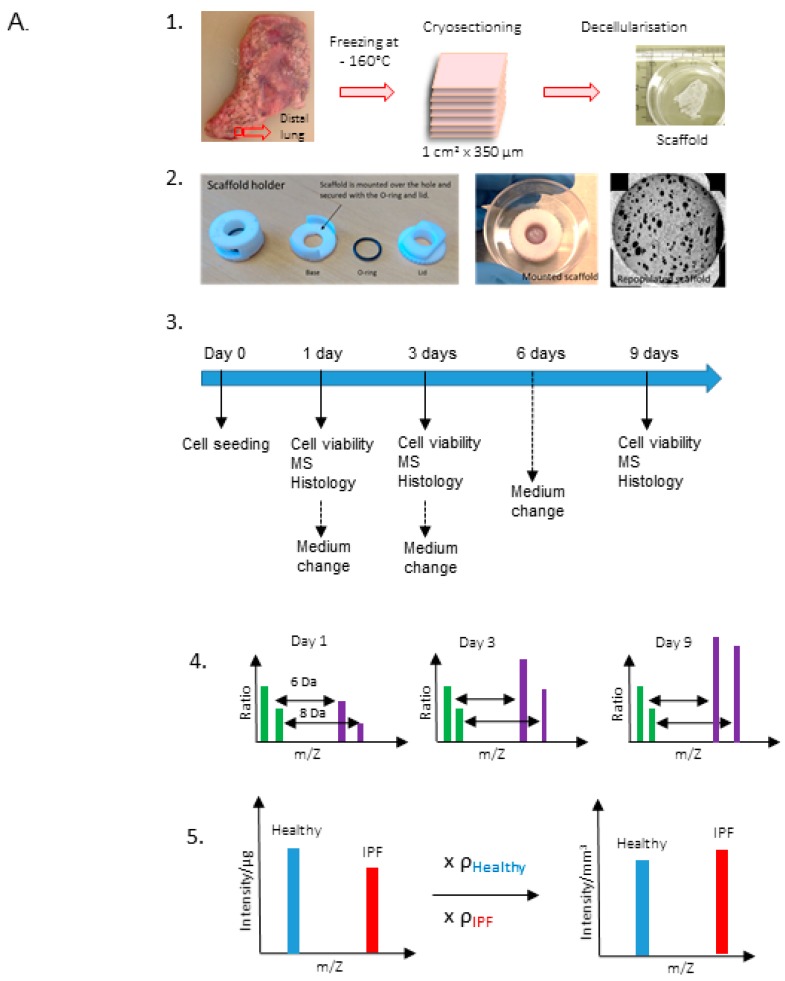

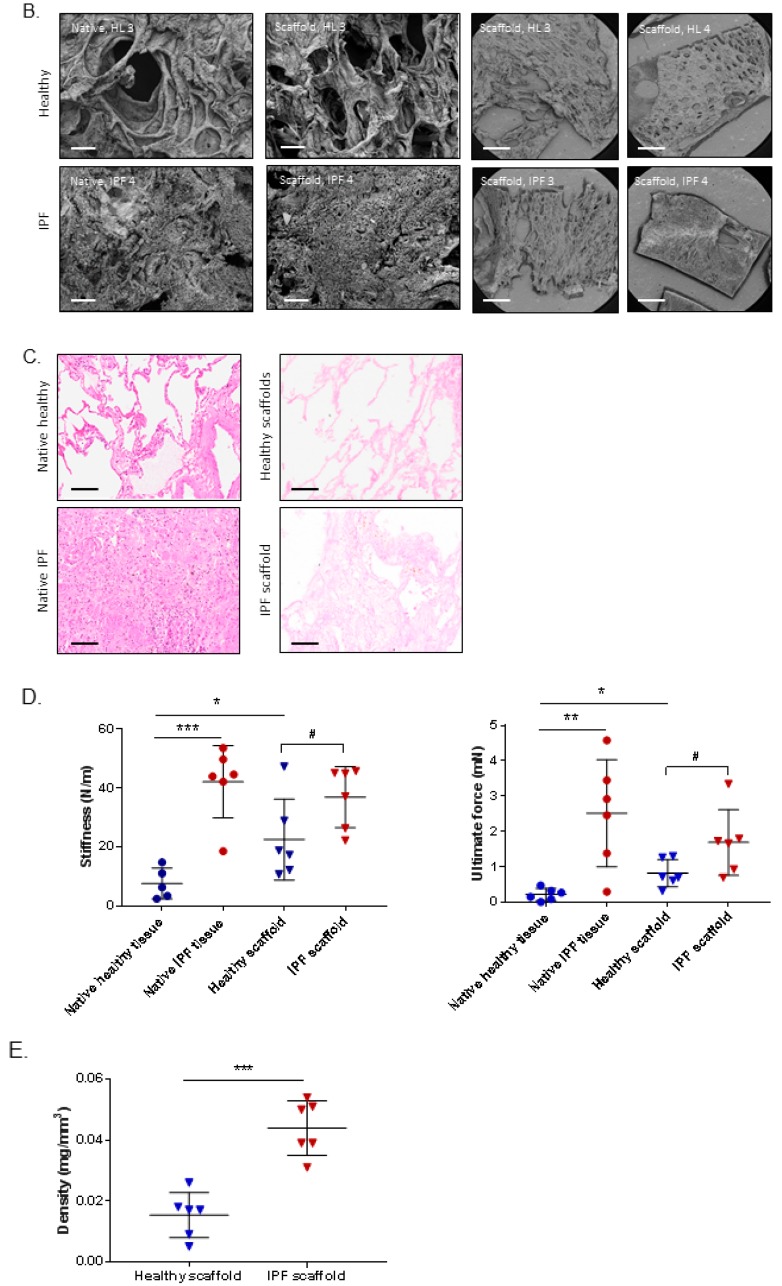
Characterization of native lung tissue and scaffolds (**A**) Schematic of experimental layout. Dissection and decellularization of 350 µm human lung tissue slices (1). Mounting of repopulated scaffolds pre-cultured in SILAC medium (2). Schematics of culture conditions and sample extractions (3). Mass spectrometry (MS) analysis on light (green bars) and heavy (purple bars) protein intensities (m/Z, protein mass/protein charge) illustrating the mass shift of 6 Da (Arg) or 8 Da (Lys) between pre-existing (scaffold extracellular matrix (ECM)) and newly produced matrisome proteins (4). Intensity/μg was adjusted for tissue density resulting in intensity/mm^3^ (5). (**B**) Representative scanning electron microscopy (SEM) images with the same magnification (scale bar = 100 μm) of native tissue (left) and decellularized tissue (scaffold) (middle) and scaffolds at an overview (right, scale bar = 1 mm) for illustration of sample variability (right). (**C**) Hematoxylin and eosin staining of native lung tissue and corresponding scaffold after decellularization of the tissue (scale bar = 100 μm). (**D**) Stiffness and ultimate force measurements of biological replicates (*n* = 3, with two technical replicates except for native healthy tissue) from native healthy and idiopathic pulmonary fibrosis (IPF) lung tissue and corresponding scaffolds (*n* = 4, with two technical replicates) derived from healthy and IPF tissue. (**E**) Density measurements of healthy and IPF scaffolds (*n* = 2, with three technical replicates). Unpaired *t*-test for significance between patient groups with *p*-values * *p* < 0.05, ** *p* < 0.01, *** *p* < 0.001. Stiffness # *p* = 0.068, Ultimate force # *p* = 0.059.

The schematic layout of the study and scaffold preparation is illustrated in Figure 1A. Macroscopic characterization of healthy and IPF lung tissue slices, using scanning electron microscopy (SEM), displayed an evident difference in tissue morphology, with a dense meshwork of matrix and compact lung architecture in IPF tissue (Figure 1B), which correspond to the end stage of long-term IPF. After decellularization, the scaffolds maintained original tissue integrity and characteristics in both IPF and healthy decellularized lung tissue, examined by SEM and in histology (Figure 1B–E). Overview images of scaffolds with SEM illustrated the heterogeneity in the IPF patient material with more or less dense tissue. The decellularized scaffolds from IPF and healthy individuals showed no signs of visible cells in the tissue, as seen with hematoxylin/eosin staining (Figure 1C). Furthermore, the cellular content was examined in decellularized IPF derived scaffolds, measuring dsDNA. In support of our previous study on healthy lung tissue by Rosmark et al. [13], dsDNA content was efficiently removed following decellularization showing only 1.5% residual dsDNA per mg tissue in IPF derived scaffolds (data not shown). In the stress–strain measurements, the native lung tissues from IPF patients showed significantly higher tensile stiffness in comparison to healthy individuals (*p* = 0.0003), as well as higher ultimate force (*p* = 0.0097) (*n* = 4) (Figure 1D). One duplicate of native lung tissue from one patient examined for stiffness was excluded and regarded as an outlier with a value (115.39) exceeding more than three standard deviations from the mean. These properties remained in the decellularized IPF scaffolds. The healthy scaffolds, on the other hand, showed a higher stiffness (*p* = 0.0485) and ultimate force (*p* = 0.0146) compared to the native tissue, although with a larger variability. Within the scaffold groups, differences in stiffness (*p* = 0.06676) and ultimate force (*p* = 0.0594) were maintained compared to difference in between native tissue groups. We did not observe any differences in stress-relaxation behavior for native lung tissue and the decellularized scaffolds for neither the healthy nor the IPF samples (Figure S1A). Force to failure curves revealed a clear shift towards higher tensile strength, with increased force to tissue displacement in IPF tissue (Figure S1B,C). Despite high patient variability, tissue density (mg/mm^3^) was significantly higher (*p* = 0.0022) in IPF scaffolds in comparison to healthy scaffolds (Figure 1E).

### 2.2. Proteomic Profiling of Lung Scaffolds

**Figure 2:**
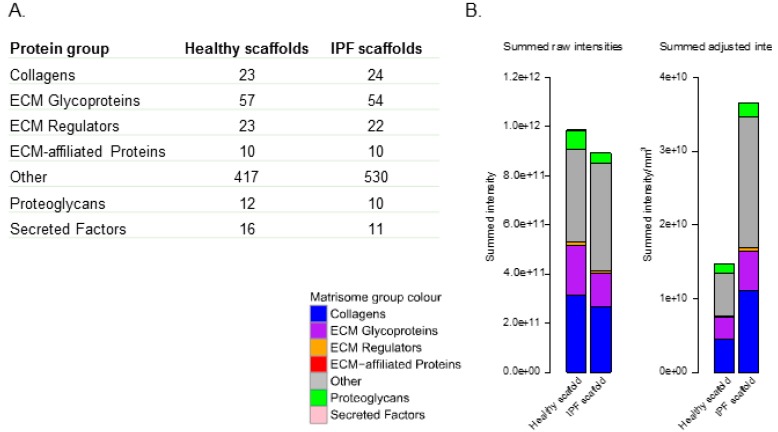

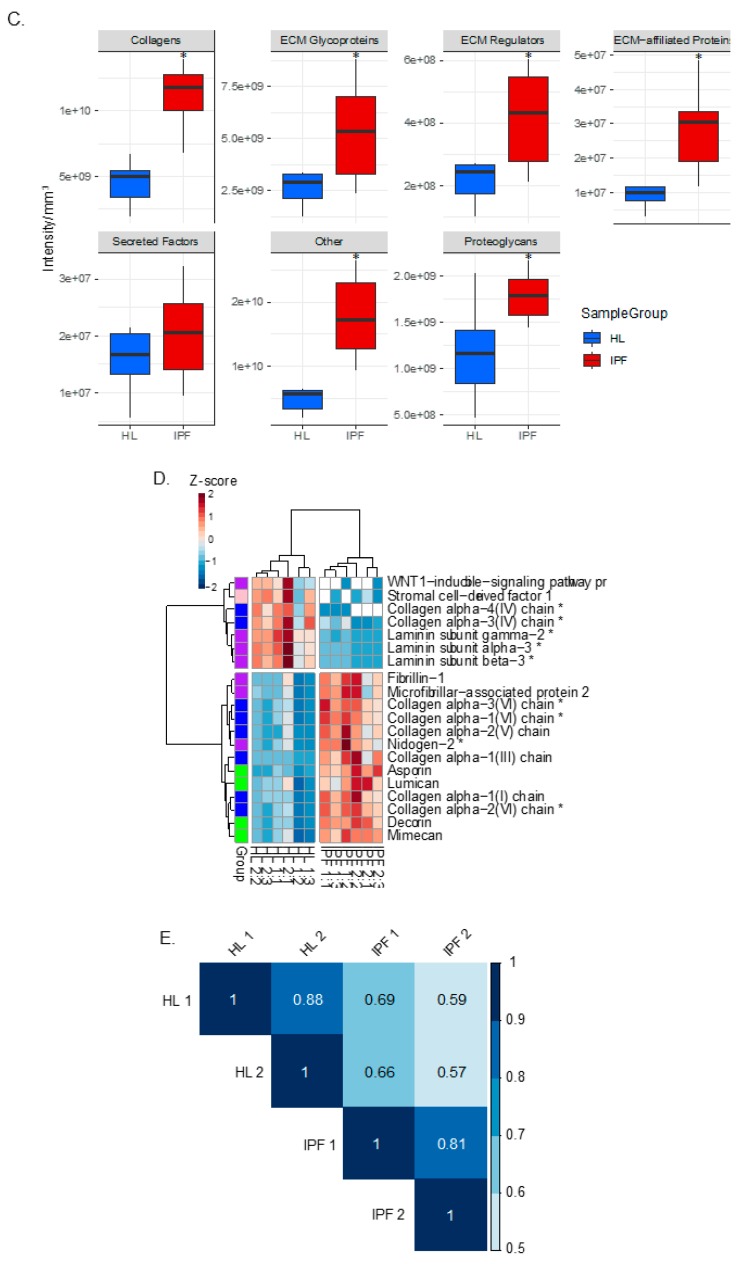

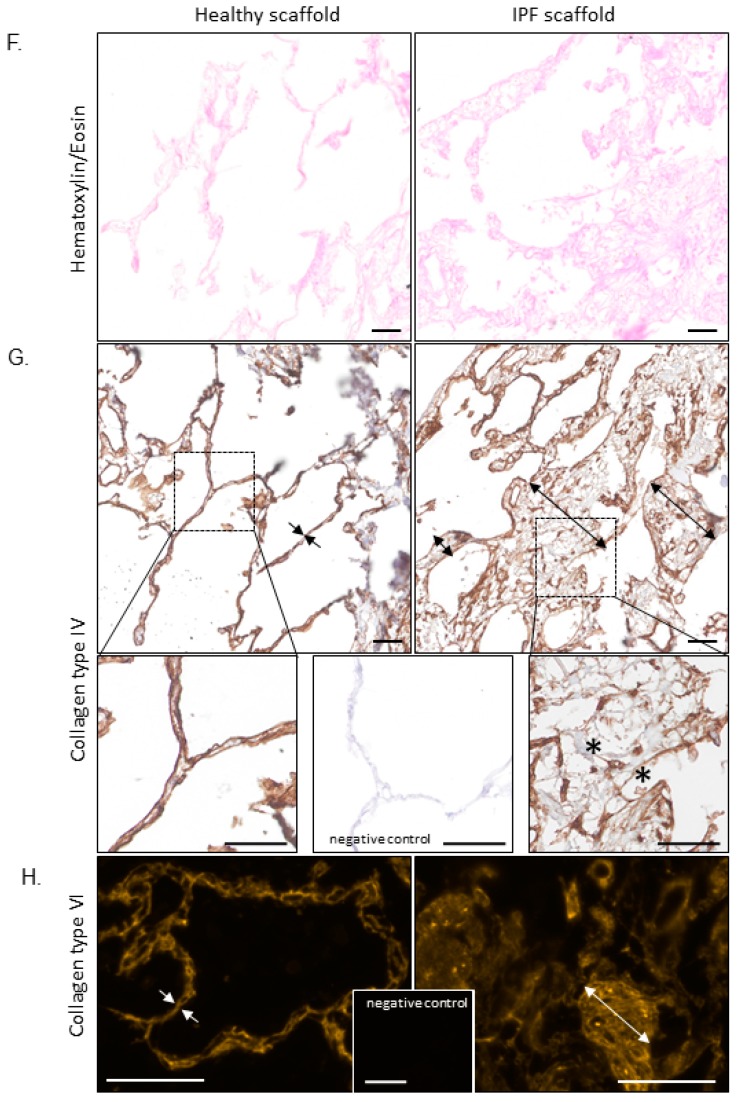
Proteomic and histological characterization of healthy and IPF derived tissue scaffolds. (**A**) Number of identified proteins in decellularized scaffolds derived from healthy individuals (biological replicates *n* = 2, technical replicates *n* = 3) and IPF patients (*n* = 2, *n* = 3). Protein groups assigned to matrisome affiliation. (**B**) Matrisome grouped summed raw intensities for proteins in healthy and IPF derived scaffolds, left panel. Matrisome grouped summed intensities after density adjustment in healthy and IPF scaffolds, right panel. Mean values for groups presented. (**C**) Statistics for summed matrisome groups. Student’s *t*-test with Benjamini-Hochberg corrected *p*-values * *p* < 0.05. (**D**) Unsupervised hierarchical clustering of significantly different matrisome proteins characteristic for the scaffold types using Z-scored values. Basement membrane proteins marked with *. (**E**) Spearman correlation between scaffold groups. (**F**) Histological verification and spatial tissue distribution of selected matrisome (or basement membrane) proteins. Hematoxylin/Eosin staining showing distinct morphological differences between thin, alveolar septa (healthy scaffold) compared to thickened, fibrotic remodeled septa (IPF scaffold). (**G**) This is accentuated (arrows) by staining for collagen type IV (brown), indicating clear basement membrane staining lining the alveolar septa in the healthy scaffold compared to disorganized fragments in the IPF scaffold, with large areas devoid of collagen type IV signal or reduced intensity (*). (**H**) Inversely, collagen type VI showed accumulation in these fibrotic structures in the IPF scaffold. Scale bar 50 µm.

In the next step, we used quantitative mass spectrometry to determine the ECM composition using a matrisome classification system [14,19,20] to investigate if the molecular composition of the scaffolds could be explained by the differences in matrisome properties between healthy and IPF scaffolds. Each group, healthy and IPF, was analyzed in triplicates from each donor, with two donors per group (Figure S2). The analysis showed protein groups containing comparable numbers of identified matrisome proteins in both healthy and IPF derived scaffolds, indicative of an equivalent protein extraction from each type of scaffold (Figure 2A). However, the number of identified non-ECM proteins (other) were higher in IPF scaffolds (530 proteins) in comparison to healthy derived scaffolds (417 proteins), a difference that could be explained by slightly increased cellular remnants in the compact decellularized IPF tissue. Nonetheless, the low content of dsDNA in IPF scaffolds verified the matrices as decellularized tissue with > 98% DNA removal [21].

The distributions of proteins in the two types of scaffolds were presented as summed intensities by matrisome groups (Figure 2B). To compensate for the discrepancy in tissue morphology between healthy and IPF derived scaffolds, the summed intensity (intensity/µg) of all proteins was adjusted for tissue density (mg/mm^3^) (Figure 1A:5). Data showed a distinct difference in intensity of matrisome groups between IPF and healthy derived scaffolds following density adjustment. The summed intensity of each matrisome group was increased in IPF scaffolds, in comparison to healthy derived scaffolds, reflecting the difference in matrisome composition. Further examination of matrisome group intensities showed a significantly higher amount of nearly all matrisome groups in IPF scaffolds compared to healthy scaffolds, seen as intensity/mm^3^ (Figure 2C). To select matrisome proteins significant for respective scaffold group we used a threshold of fold change 2 and a Benjamini-Hochberg corrected *p*-value below 0.05 between the healthy and diseased group. Visualization of significantly different matrisome proteins using unsupervised hierarchical clustering of Z-scored values, identified matrisome proteins characteristic for healthy and IPF derived scaffolds respectively (Figure 2D). The top cluster in the heatmap depicts matrisome proteins that were less abundant in IPF scaffolds, while the bottom cluster showed proteins more abundant in IPF scaffolds, as compared to healthy scaffolds (Figure 2D). Despite recognized lung tissue heterogeneity coupled to IPF patients [22] and limited number of patient samples, IPF derived scaffolds showed high correlation in matrisome composition, seen both in biological replicates and in between donors (rank = 0.81) (Figure 2E). Healthy derived scaffolds showed similar matrisome correlation (rank = 0.88) within its group. Further examination of the significantly expressed matrisome proteins showed that nine out of 20 of them were assigned as BM associated proteins (Figure 2D) [20]. Nidogen-2 and Collagen type VI (α 1,2,3 chain) were clustered together as more abundant in IPF scaffolds in comparison to healthy scaffolds, whereas laminin γ2, laminin β3, laminin α3 and collagen type IV (chains α3 and α4) were significantly decreased in IPF scaffolds. Hematoxylin/eosin staining showed distinct morphological differences with thin alveolar septa in the healthy scaffolds compared to thickened and remodeled septa in the IPF scaffolds (Figure 2F). This was also clearly illustrated by immunohistochemistry (IHC) for collagen type IV (Figure 2G) and type VI (Figure 2H). In the healthy lung scaffold, the BM of both sides of the alveolar septum displayed a thin, even double-line indicating the presence of collagen type IV. In the IPF lung scaffold however, this staining was uneven and often poorly defined, with areas of decreased staining intensity and both thickened and thinned BM structures. Most obvious was the increased alveolar septum thickness, representing large areas without collagen type IV signal. Inversely, collagen type VI showed accumulation in exactly these fibrotic structures in the IPF scaffold. Furthermore, the IPF scaffolds showed disorganized BM-fragments and possibly increased microvasculature. In summary, this illustrates the loss of normal lung organizations in IPF with altered BM membrane composition and structure.

### 2.3. Repopulated Scaffolds

**Figure 3:**
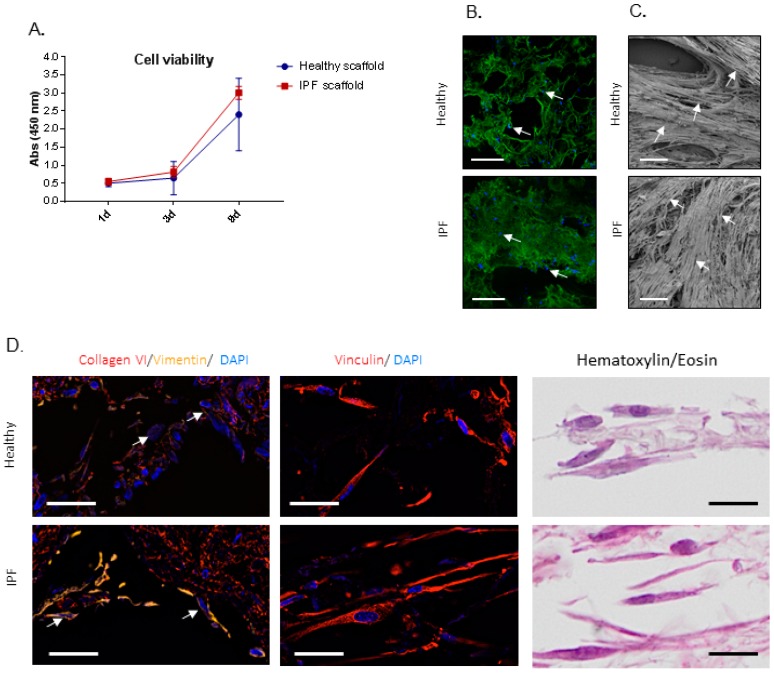
Cell viability and cell attachment of repopulated IPF and healthy tissue scaffolds. (**A**) Cellular viability of primary lung fibroblast repopulated on tissue scaffolds (biological replicates *n* = 4), shown as mean ± SD. (**B**) Confocal live imaging after 1 day of culture, showing equal cell attachment. Cell staining (blue), autofluorescent scaffold (green). Arrows indicate cells. Scale bar = 500 µm. (**C**) SEM after 9 days of culture, visualizing cellular differences in orientation in the scaffolds and repopulation. Arrows indicate elongated cells. Scale bar = 100 µm. (**D**) Visualization of repopulated scaffolds, showing fibroblasts attaching to the surrounding tissue (here: collagen type VI) and cytoskeleton (vimentin) and patterns of focal adhesions (vinculin). Hematoxylin/eosin staining of corresponding scaffolds shown in the right panel. Arrows: different intensities of vimentin in fibroblasts repopulating healthy vs. IPF scaffolds. Scale bar = 20 µm.

After characterization of scaffold properties, we continued to study the cellular response of primary human lung fibroblasts cultured up to 9 days on scaffolds derived from IPF patients and healthy individuals. For these experiments we seeded fibroblasts, derived from a healthy donor, on scaffolds derived from four patients for each group and cultured in duplicates for each patient (*n* = 4 per group). To start, we examined if cellular attachment and viability varied between the two types of scaffolds. Cellular viability, measured as metabolic activity, showed no difference between the two types of scaffolds after 1 day of culture, indicative of equivalent numbers of attached cells (Figure 3A), also visualized by confocal microscopy (Figure 3B).

This result was also confirmed by counting the number of cells left in the wells after cell seeding, where no difference between the groups was seen. No significant difference in cellular viability was detected in any of the scaffolds measured up to 9 days in culture (Figure 3A). SEM imaging showed differences in cell orientation between the groups, with cells densely packed on top of dense areas of the IPF scaffolds and heavily repopulated less dense structures, whereas cells cultured on healthy derived scaffolds followed and maintained open lung structures (Figure 3C). To visualize cellular attachments and organization in the scaffolds, repopulated scaffolds were antibody labeled for collagen type VI, a cell binding protein, in combination with the mesenchymal cell marker vimentin and the focal adhesion protein vinculin (Figure 3D). Results showed similar staining of collagen type VI in both types of scaffolds, again representing thin alveolar septa in the healthy scaffold compared to heavily remodeled parenchyma in the IPF scaffold. Interestingly, repopulating fibroblasts on IPF scaffolds appeared to show more intense vimentin staining compared to cells on healthy scaffolds, which is in line with other studies demonstrating a correlation between vimentin and substrates stiffness [23]. Furthermore, cells on IPF scaffolds were primarily situated in less dense tissue areas and appeared stretched and elongated, lining surface edges of pulmonary structures (Figure 3D). To further examine cellular attachments, immunofluorescence (IF) staining for vinculin was performed, an integrin involved in intracellular signaling [24] which showed no difference in cellular distribution between healthy and IPF derived scaffolds.

### 2.4. Proteomic Profiling of Matrisome Proteins in Repopulated Healthy and IPF Lung Scaffolds

**Figure 4:**
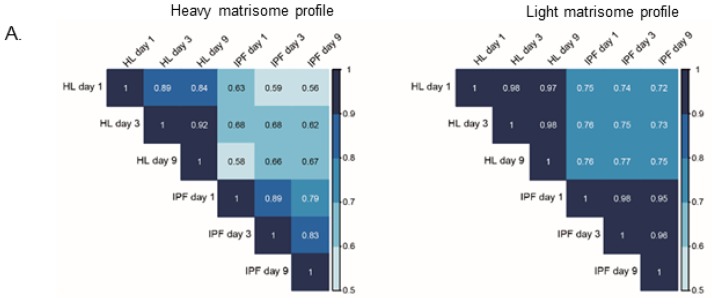

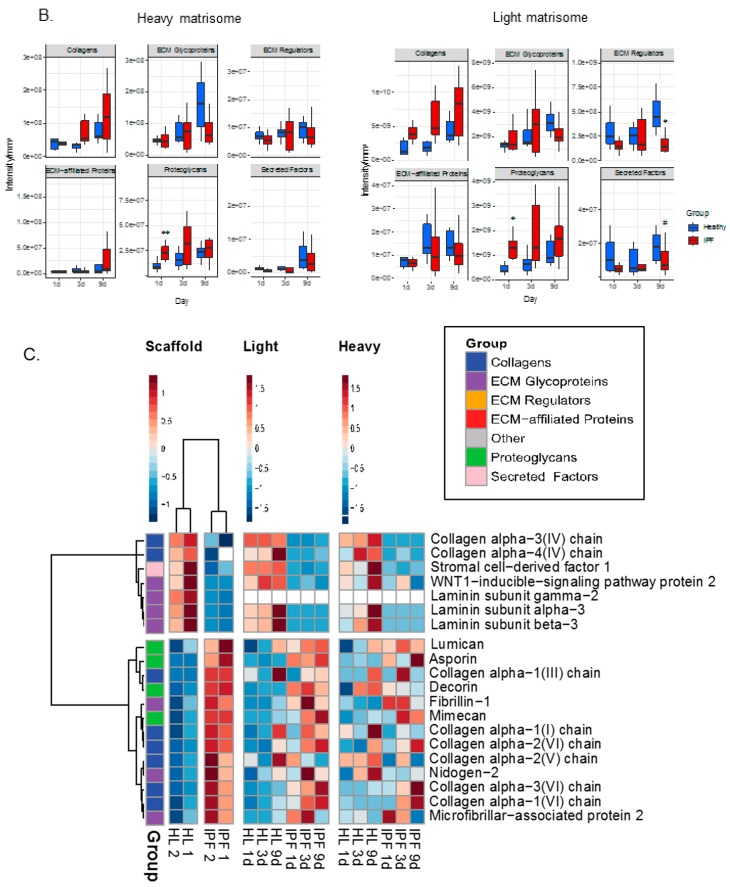
Proteomic characterization of matrisome proteins in tissue scaffolds repopulated with SILAC labelled fibroblasts. (**A**) Spearman correlations of matrisome proteins in repopulated healthy and IPF scaffolds at day 1, 3, and 9 after repopulation. Scaffold group mean MS-intensities for each time point presented (biological replicates *n* = 4, technical replicates *n* = 2). (**B**) Statistics for matrisome groups for repopulated healthy and IPF scaffolds over time calculated from summed matrisome groups. Student’s *t*-test with Benjamin–Hochberg corrected *p*-values for significance between patient groups of the same time point with *p*-values * *p* < 0.05, ** *p* < 0.01. Light # *p* = 0.089 (Secreted Factors). (**C**) Heatmap of matrisome proteins over time. Unsupervised hierarchical clustering of Z-scored values (ward.D2). Scaffolds are presented as patient means (biological replicate *n* = 2) and repopulated scaffolds (light and heavy) are presented as patient group mean intensities for each time point (*n* = 4) with technical replicates for each group (*n* = 2). Light and heavy intensities were selected and visualized according to previous scaffold clustering.

Healthy primary human fibroblasts were cultured in SILAC-medium 5 days before cellular seeding and over the whole culture period on the scaffolds. In this experiment the cells take up heavy amino acids from the media and start to produce proteins with heavy amino acids that are distinguishable in the mass spectrometer from residual scaffold proteins that only contain light amino acids (Figure 1A). This enabled us to follow protein turnover over time from day 1, by differentiating between newly synthesized cell-derived proteins (heavy) and pre-existing matrisome proteins in the scaffold (light). As with the mass spectrometry (MS) data for the decellularized scaffolds, the data for the repopulated scaffolds was adjusted for differences in tissue density and a mean value was calculated for each group at each time point. Density adjustment allowed us to study an equal tissue volume and thereby the same number of cells in the two types of scaffolds. The matrisome protein differences between the groups over time were analyzed through a Spearman correlation test, which clearly demonstrated that newly synthesized proteins (Figure 4A, heavy) from cells cultured on IPF scaffolds had a different protein composition compared to healthy individuals. Scaffolds in the IPF group correlated within its group over time as did the healthy individuals (Figure 4A, light). The temporal changes of overall matrisome compositions for each type of scaffold were shown as heavy and light matrisome protein groups over time (Figure 4B). Interestingly, fibroblasts diverged in their production (heavy intensity/mm^3^) of matrisome proteins, detected as early as day 1 of culture on IPF derived scaffolds. At day 1, the fibroblasts produced a significantly (*p* = 0.0069) higher level of proteoglycans compared to cells cultured on healthy scaffolds (Figure 4B). Over time, we observed a tendency of increased collagens production in repopulated IPF scaffolds, however the level of ECM glycoproteins remained unchanged. Examination of the preexisting scaffold composition (light intensity/mm^3^) (Figure 4B) representing ongoing ECM remodeling, showed increased amounts of proteoglycans (*p* = 0.0231) such as perlecan and lumican in IPF scaffolds at day 1 (Figure S3). However, at day 9, IPF scaffolds showed significantly decreased amounts of ECM regulators (*p* = 0.029) (Figure 4B) e.g., TIMP-3 (Figure S3) and secreted factors (*p* = 0.089) (Figure 4B).

To exclude cell number variabilities in between the two types of scaffolds after adjusting the values, we examined heavy and light labeled histones in each group over time, showing no significant difference between IPF and healthy repopulated scaffolds at any time point (data not shown). These results indicate that the identified diversity in protein synthesis was dependent on the original scaffold properties and not by variety in cellular content. To further describe this finding, we selected the matrisome proteins found to be significantly different and descriptive for the respective decellularized scaffold group, and analyzed these further showing temporal differences in repopulated scaffolds. We compared both newly synthesized matrisome proteins (Figure 4C, heavy) as well as changes in the original scaffold composition over time (Figure 4C, light) based on the significantly different matrisome proteins in the scaffolds from the starting point (Figure 4C, scaffold). The top cluster in the heat map presents the matrisome proteins that are more abundant in healthy scaffolds in comparison to IPF scaffolds. Within its own group, newly synthesized proteins from IPF and healthy scaffolds showed similar protein expressions over time (top cluster). The overall pattern of newly synthesized matrisome proteins appeared to overlap with the original scaffold composition representative for each group. These results indicate that the characteristics of the original scaffolds can influence cellular activity, stimulating scaffold specific protein production in primary fibroblasts, thus mimicking the composition found in the original scaffold.

**Figure 5:**
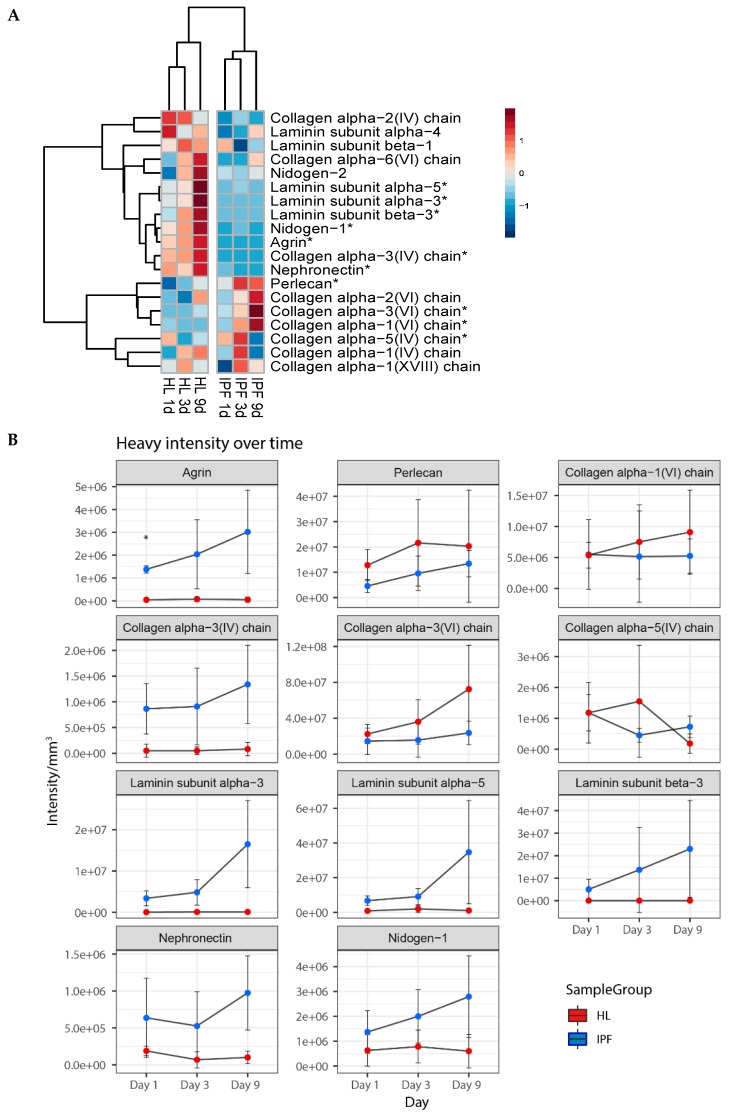

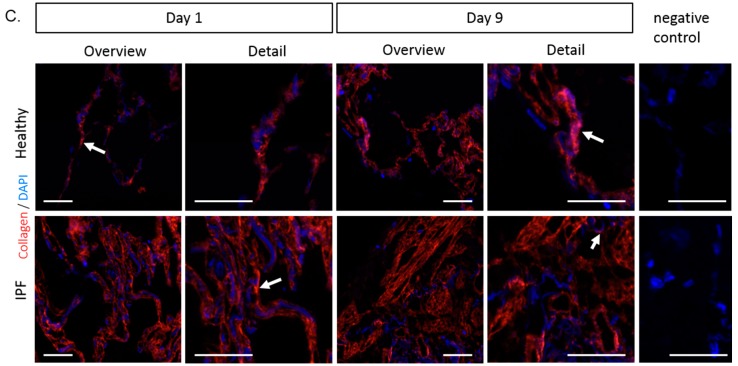
Synthesis of basement membrane proteins in repopulated scaffolds. (**A**) Heatmap of basement membrane (BM) proteins over time. Repopulated scaffolds as group mean heavy intensities for each time point (biological replicates *n* = 4, technical replicates *n* = 2). With Student’s *t*-test, significantly differentially expressed proteins are marked with *. Perlecan = Basement membrane-specific heparan sulfate proteoglycan core protein. Production of significantly different basement membrane proteins in repopulated IPF and healthy scaffolds. Protein intensity shown as mean heavy intensity (**B**) over time for each group with SD. Student’s *t*-test with Benjamin–Hochberg corrected *p*-values for significance between patient groups of the same time point with *p*-values * *p* < 0.05. Blue = Healthy, Red = IPF. (**C**) Antibody labeling of repopulated scaffolds, at day 1 and day 9 of culture, showing collagen type VI (α1) (red) with DAPI staining (blue). Images of collagen VI staining, illustrate newly synthesized protein and original scaffold composition, with arrows exemplifying positive staining. Scale bar = 50 µm.

Furthermore, to connect to our previous findings in the decellularized scaffolds, we examined how BM protein production was affected over time in each type of scaffold (Figure 5A). Most of the significantly different expressed BM proteins showed a reduced expression in IPF repopulated scaffolds (top cluster, Figure 5A) as compared to healthy scaffolds. Changes in protein intensity were analyzed over time and significantly differently expressed BM proteins (11 out of 20 BM proteins) were presented as mean heavy intensity for each group (Figure 5B). Interestingly, healthy scaffolds showed an increased production of nidogen-1 and laminins (subunit α3, β3, and α5) over time, whereas in the IPF scaffolds the synthesis was low or undetected. Similar responses were seen with collagen IV production of α3 and α4 chains. For repopulated IPF scaffolds we found an increased production of the following structural BM proteins; basement membrane-specific heparan sulfate proteoglycan core protein (perlecan), collagen type VI chains α1, α2, and α3. The diverse expression and downregulation of several heavy labeled BM proteins in repopulated IPF scaffolds further supports the manifestation of a disorganized BM as previously visualized in IPF derived scaffolds. Scaffold changes over time were shown with light labeled protein intensity, showing several BM proteins with similar temporal patterns as heavy intensities (Figure S4).

Based on the quantitative data in Figure 5B and Figure S4, we show with antibody labeling, the spatial expression pattern of collagen type VI in repopulated scaffolds, showing an intensified expression level at day 9 in healthy scaffolds as compared to day 1 (Figure 5C). In IPF scaffolds, collagen VI appeared stable over time showing no distinct visual difference in antibody labeling. The overall expression of collagen VI appeared visually increased in IPF scaffold vs. healthy, which could be explained by the higher density of the tissue.

**Figure 6:**
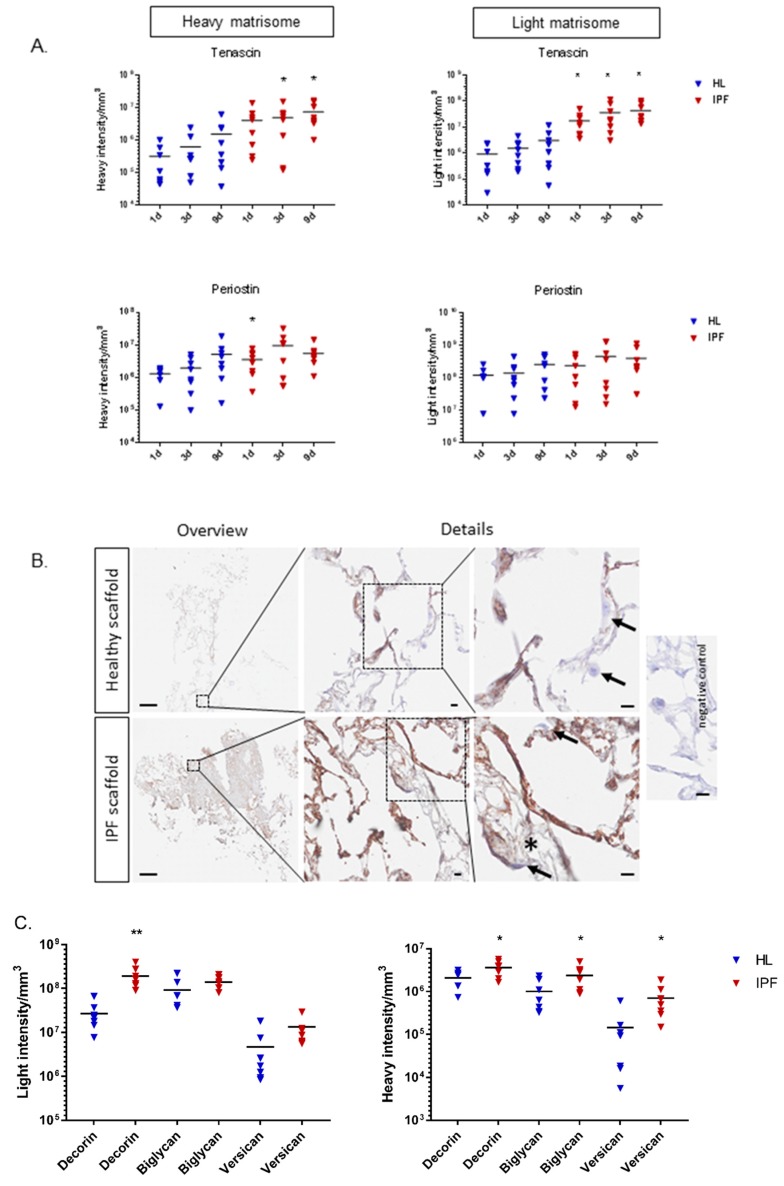

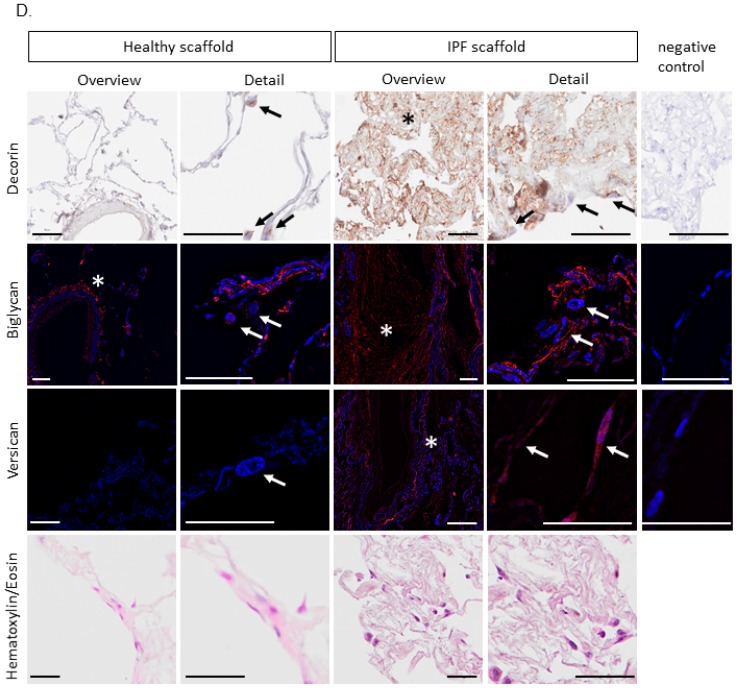
(**A**) Graphs of heavy and light intensity/mm^3^ of tenascin and periostin of repopulated scaffolds (biological replicates *n* = 4, technical replicates *n* = 2) with calculated grand mean. Student’s *t*-test for significance between patient groups means for each time point (* *p* < 0.05). (**B**) Periostin in repopulated scaffolds (day 1). Antibody labeling indicating periostin (brown) in certain areas in alveolar septa in the healthy scaffolds, and to a stronger degree in less remodeled tissue areas in the IPF scaffolds. Largely absent periostin staining in heavily remodeled IPF tissue areas (*). Arrows highlight differences in intracellular staining. Scale bar overview 500 µm, details 10 µm. (**C**) Graphs of original (light) and newly synthesized (heavy) proteoglycans decorin, biglycan, and versican of repopulated scaffolds (biological replicates *n* = 4, technical replicates *n* = 2) are presented with calculated grand mean of light and heavy intensity at day 1. Student’s *t*-test for significance between patient groups (* *p* < 0.05, ** *p* < 0.01). (**D**) Antibody staining illustrating proteoglycan increase in IPF vs. healthy scaffolds (day 1) of newly synthesized proteoglycans including original scaffold composition. Decorin (brown): IPF scaffold showing intrinsically more decorin, with cellular signal in fibroblasts on both scaffold types. Biglycan (red): rare intrinsic biglycan in healthy scaffolds apart from vessels, but strong accumulation in IPF scaffolds. Cellular signal of biglycan in fibroblasts on both scaffold types were seen. Versican (red): absent intrinsic versican in healthy scaffolds, but distinct accumulation in IPF scaffolds. Prominent cellular signal in fibroblasts on IPF scaffolds, but not on healthy scaffolds. * extracellular deposition in repopulated scaffolds. Arrows highlight differences in intracellular staining. Corresponding hematoxylin/eosin staining of the repopulated scaffolds shown in bottom row. Scale bar 50 µm.

At further examination of heavy labeled proteins, we identified that the synthesis of tenascin and periostin was significantly altered in repopulated IPF scaffolds, matrix components that have been associated with the progression of IPF [25,26]. Tenascin and periostin were also found to be elevated in the original scaffold composition of IPF (Figure 6A). Fibroblasts on IPF scaffolds produced significantly higher amounts of tenascin (*p* = 0.044 at day 3, *p* = 0.027 at day 9) and periostin (*p* = 0.039 at day 1) (Figure 6A), protein expression patterns that have been implicated in fibrosis [25,27,28]. IHC staining of repopulated scaffolds visualized periostin distribution (Figure 6B). Periostin was found in certain areas of the thin alveolar septa in healthy scaffolds. IPF scaffolds, on the other hand, had a stronger staining in less remodeled areas and very low periostin signal in the heavily remodeled and fibrotic tissue areas. No apparent intracellular periostin could be detected by IHC.

Synthesis of the proteoglycan decorin was upregulated (*p* = 0.043) in fibroblasts cultured on IPF scaffolds on day 1 of repopulation (Figure 6C). Decorin labeling showed clear intracellular and periocellular staining with a general enhanced overall tissue expression in IPF derived scaffolds (Figure 6D). Interestingly, these early changes in proteoglycan production were also detected for biglycan (*p* = 0.040) and versican (*p* = 0.027), showing significantly increased levels in repopulated IPF derived scaffolds (Figure 6C). The increased production of proteoglycans was further supported with antibody labeling (Figure 6D). IPF scaffolds showed higher levels of decorin compared to healthy scaffolds, and intracellular staining of decorin in fibroblasts were found on both scaffold types. Biglycan staining showed a strong intrinsic accumulation in IPF scaffolds, whereas healthy scaffolds only had sporadic staining apart from vessels. Intracellular biglycan could be found in fibroblasts cultured on both scaffold types. As for versican, staining was largely absent in healthy scaffolds, but a distinct intrinsic accumulation in IPF scaffolds could be seen as well as prominent cellular signal in fibroblasts cultured on IPF scaffolds. No intracellular staining of versican could be seen in cells cultured on healthy scaffolds.

In summary, these results demonstrate that the material properties of the ECM affected fibroblast activity, thus supporting a profibrotic phenotype when cultured in a diseased milieu.

## 3. Discussion

The ECM has important biological functions such as regulating wound healing responses and tissue remodeling through cellular interactions [7,29]. The implementation of decellularized IPF and healthy lung tissues as scaffolds represents a promising approach to study the biological function of the ECM and how vital changes in matrisome properties, both in composition and biomechanically, influence cell behaviors [12]. In this study, we performed an in-depth characterization of the structural properties of these acellular lung matrices derived from healthy individuals and IPF patients, with regards to morphology, tissue density, and stiffness. Alterations of these important features, linked to the pathophysiological changes seen in IPF scaffolds, were sustained following decellularization. IPF is thought to be the result of an aberrant wound healing process involving abnormal deposition of matrix proteins e.g., collagens [1,30], and as seen in this study leading to almost a three-fold increase in tissue density compared to healthy, accompanied by a five-fold and 60% increase in stiffness for native IPF tissue and IPF scaffolds, respectively. Decellularization seemed to solely affect the biomechanical properties of healthy scaffolds. Essentially, collagen content is retained after the decellularization process in healthy lung tissue, while elastin content is affected to some degree and even more the proteoglycans along with the ECM glycoproteins [31]. Our data suggest that removal of these charged proteins most likely led to changes in biomechanical properties due to lost electrostatic interactions, leading to an entangled micro-structure, ultimately increasing the tensile strength of healthy scaffolds, which might influence cell-matrix interactions. The IPF scaffolds, on the contrary, had a higher content of collagens compared to healthy scaffolds and a largely absent BM along with the removal of surfactant proteins, which gives specific biomechanical properties of the scaffolds. Consistent with morphological differences of more or less fibrotic scaffold samples, the biomechanical properties of the scaffolds demonstrated large variations. Despite this, both within IPF patients and within biological replicates, the IPF scaffolds were distinctly separated from healthy scaffolds, actually exhibiting rather homogenous tissue characteristics, strengthening the results from our limited number of biological replicates (Figure S5).

One of the important findings was that there were differences in the abundance of distinct matrisome proteins between healthy and IPF scaffolds, with nine out of 20 of these being BM components. Visualized with collagen type IV antibody labeling, the BM showed large spatial differences between healthy and IPF scaffolds. The loss of BM integrity of the alveolar-capillary membrane along with an accumulation of collagen type VI, without normal structure reconstruction, causes an abnormal lung architecture, thought to promote fibrosis [30], where fibroblasts and especially myofibroblasts are known to be the main matrix producers and key players in fibrosis [4]. With that in mind, our aim was to examine how changes in ECM properties affected cellular responses in IPF. When IPF scaffolds were repopulated with healthy fibroblasts, we demonstrated a significantly reduced production of important BM complexes such as nidogens, laminins, and collagen IV in IPF scaffolds, results that are in support of a previously reported study [32]. In our study, laminin α3, α5, and β3 were not produced at the same level in IPF scaffolds as in healthy scaffolds. Data, which is in line with an in vivo study, where the loss of laminin α3 augmented the progression of lung fibrosis, is suggestive of its contribution to IPF disease progression [33]. Underlying the BM are anchoring matrix components perlecan and collagen type VI chains α1 and α3, which were elevated in repopulated IPF scaffolds, indicating an imbalance in ECM turnover with a build-up of matrix underlying the fragmented BM. Furthermore, an early induction in synthesis of proteoglycans decorin, lumican, biglycan, and versican (Figure 6C, Figure S3), as well as the ECM regulator, TIMP-3, (Figure S3) in IPF scaffolds compared to fibroblasts cultured on healthy scaffolds further strengthen the picture of a promotion of a profibrotic feedback loop. Proteoglycans are multifunctional proteins involved in wound healing responses and shown to be elevated in lungs from IPF patients [34,35,36,37]. Fibroblasts from lung fibrosis patients have shown an increased production of small proteoglycans, with decorin as the major proteoglycan produced with implications in pulmonary fibrotic responses [35,38]. These findings were replicated in our study and further support the hypothesis that fibroblast activity is modified by certain elements of the ECM.

In a previous study, where primary fibroblasts were cultured on healthy lung scaffolds freely floating in culture medium [13], cells contracted the surface area of the scaffold from approximately 1 cm^2^ to 1 mm^2^ in less than 9 days. To prevent this and to more closely mimic the physiological conditions, we introduced custom-made holders to mount the scaffolds in order to sustain a stretched and organized lung tissue structure during cell culture. This approach clearly demonstrated the importance of imposing a static stretch of the scaffolds, sustained by the holders, to transduce forces similar to the native situation. It has been shown that cells sense resistance to pulling as well as the local environment due to protein conformation, substrate rigidity, and architecture [39]. Repopulated scaffolds showed equivalent numbers of cells attached in both healthy and IPF scaffolds, verified by cellular viability and histone levels over time. In addition, patterns of focal adhesions, shown with vinculin staining, did not appear to be different in the groups. Although, the cell morphology appeared to be similar in both types of scaffolds, fibroblasts on IPF scaffolds seemed to have a higher accumulation of vimentin, indicating a shift in the cellular response due to an increased stiffness, also seen by others [40]. Compositional alterations of the ECM affect mechanical properties of tissues, which in turn influence how the cells perceive its local environment in terms of forces and ECM tension through integrin-ECM interactions that in turn will have an impact on the intracellular signaling [24,39]. An enhanced matrix stiffness with reduced tissue compliance is known to promote fibroblast activation and fibrosis [41]. We demonstrated an increased tissue stiffness in IPF scaffolds, as recognized in other studies of native lung tissue from IPF patients [42], which in turn had an effect on fibroblast activity. The stiffness of the ECM is increased in areas of fibrosis [43] and stimulates fibroblast migration, differentiation, and other cellular events that are associated with tissue remodeling [6]. Invasive migratory fibroblasts degrade and disrupt surrounding barriers to propagate its migration toward stiff and fibrotic areas of the lung [44]. In our study, repopulated IPF scaffolds synthesized lower amounts of the MMP (metalloproteases) inhibitor TIMP-3 (Figure S3). The shift in ECM regulators connect to our previous study by Ahrman et al. and also to other studies showing decreased levels of tissue inhibitors and increased levels of proteases in IPF [8,10,45]. Deprived balance of MMPs and TIMPs, enzymes necessary for matrix reorganization, contribute to a pathological turnover rate of the ECM [45,46]. These factors with both direct and indirect regulation of ECM structures, including activation of growth factors, cytokines, and chemokines, have been suggested to have an important role in the development of fibrosis, however, with diverse and complex functions [47]. We also saw that ECM regulators and secreted factors decreased at day 9 only in the IPF light matrisome, indicative of a high enzymatic activity in fibroblasts cultured on IPF scaffolds, thus resulting in enhanced release and/or removal of ECM components to the medium compared to fibroblasts cultured on healthy scaffolds. We saw that the fibroblasts filled up the spaces in less dense areas and covered dense areas with a compact cell sheet in the IPF scaffolds, while the alveolar structure was maintained in the healthy scaffolds. This feature may be explained by the loss of an intact BM in combination with an increased stiffness in the IPF scaffolds. Collectively, these characteristics may contribute to a dysregulated and imbalanced proteolysis of matrix proteins, changes that we saw in the temporal expression of light labeled BM proteins in between the two types of scaffolds (Figure S4).

Interestingly, when cultured on IPF scaffolds, fibroblasts showed an increased production of tenascin and periostin, proteins which are upregulated in IPF patients, with the latter recognized as a disease marker for IPF progression [25,26]. Tenascin is a large ECM glycoprotein transiently expressed during wound healing and involved in several tissue remodeling processes, which was reflected in our system where the fibroblasts responded to the altered milieu in the IPF scaffolds. This protein stimulates migration of fibroblasts and increased mechanical stiffness, seen in vitro, and is upregulated in patients with IPF, especially at fibroblastic foci [26,27,48]. The matricellular protein periostin is able to bind to tenascin facilitating its incorporation to the ECM [49]. In seemingly healthy looking areas in the IPF scaffolds we found strong staining for periostin, whereas in heavily remodeled and fibrotic areas the staining was absent. The spatial distribution of periostin may direct the progression of fibrosis by acting as an early trigger for matrix build-up, seen with an initial high production in IPF scaffolds compared to healthy, which supports our observation that fibroblast migrate to less dense areas in IPF scaffolds.

**Figure 7:**
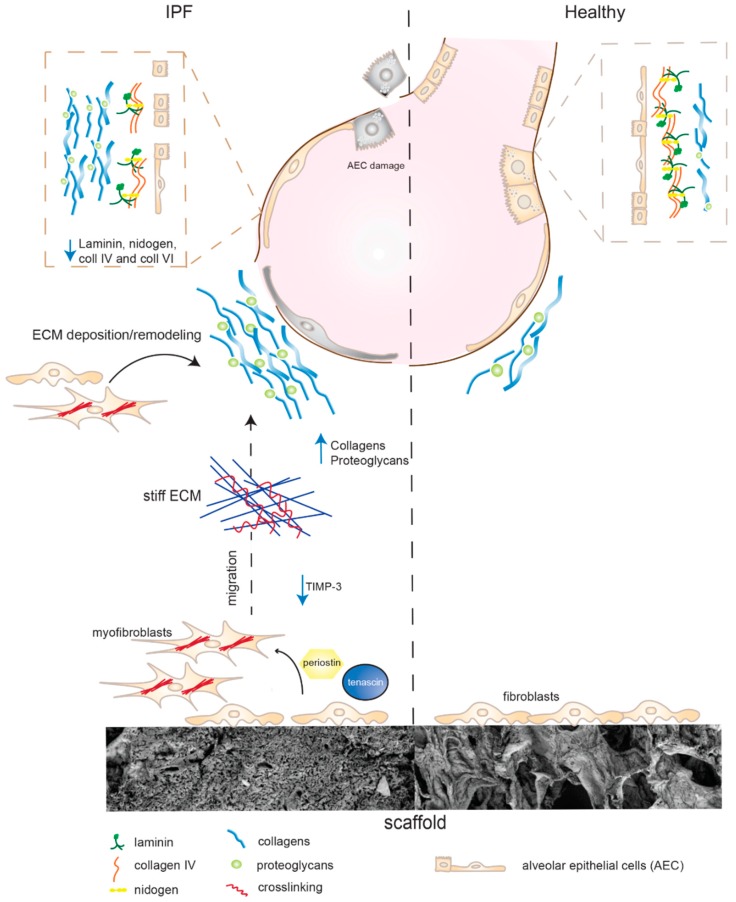
Cell-matrix interactions in IPF. Fibroblasts cultured on stiff IPF scaffolds secrete increased amounts of periostin, known to stimulate myofibroblast differentiation and migration. Increased synthesis of tenascin and reduced levels of metalloprotease inhibitors (TIMP-3) support migration and movement toward stiffer tissue. Fibroblasts become activated and generate increased deposition and build-up of collagens and proteoglycans (decorin, versican, and biglycan). Alveolar epithelial cell (AEC) damages causes basement membrane disruption and the loss of structural barriers. Fibroblasts on IPF scaffolds reduced their production of BM complexes (laminins, nidogens, and collagen IV), potentially hindering the rebuild of a functional BM for anchoring AEC.

In accordance with previous transcriptome studies by Parker et al. [29], our results support the notion of the ECM being a key driver and regulator of fibrosis, causing a positive feedback loop between fibroblasts and the diseased ECM (Figure 7), which warrants further investigation. We hypothesized that the biomechanical properties and the composition of the ECM dictate the cellular response in human primary fibroblasts as reflected by the overall cellular response to a healthy and diseased matrix.

We saw that fibroblasts cultured on stiff IPF scaffolds secreted increased amounts of periostin, known to stimulate myofibroblast differentiation and migration. This in combination with an increased synthesis of tenascin and reduced levels of metalloprotease inhibitors (TIMP-3) supports the migration of fibroblasts towards a stiffer ECM. The cells become activated and generate increased deposition and build-up of collagens and proteoglycans including decorin, versican, and biglycan. With the elevated levels of periostin, the incorporation of tenascin-C into the matrix may be facilitated as well as the formation of collagen fibrils, assisted by decorin. The IPF scaffolds have a clear disruption of the BM, which is thought to arise from alveolar epithelial cell (AEC) damages leading to the loss of structural barriers. This may promote transdifferentiation of epithelial cells to mesenchymal cells which in addition activates the progression of remodeling. Interestingly, fibroblasts on IPF scaffolds reduced their production of BM complexes (laminins, nidogens, and collagen IV), potentially hindering the rebuild of a functional BM for anchoring AEC. The continuation of fibroblast activation in combination with a disorganized BM seem to propagate changes in the matrisome properties, further promoting disease progression.

This study is separate from previously performed studies on pulmonary fibrotic lung tissue [12,29] as we focused on the distal lung properties of IPF, where this disease typically manifests itself with subpleural fibrotic formations. Furthermore, the advantage of our human 3D-model excludes the effect of resident cellular components of the parenchymal tissue characteristics and focuses on the cellular response of the ECM.

We have clearly demonstrated that the biomechanics and the matrisome composition of the IPF scaffolds are closely connected, which make up an intricate biological system controlling cellular behavior with the ability to sustain a profibrotic lung environment. To mimic the physiological conditions more closely and to maintain the complex structure of decellularized lung tissue during repopulation, a novel approach was implemented in this study through the application of scaffold holders, manufactured to mount lung tissue in order to impose a static stretch. By combining the biomechanical properties of a scaffold, linked with its own proteomic profile, unique matrisome properties were identified in IPF patients in comparison to healthy individuals. In a novel way of analyzing proteomic data, tissue density adjustments enabled an in-depth study of ECM turnover in IPF and healthy scaffolds by recognizing structural heterogenetic differences, which thereby separated the two types of tissues. The cellular responses studied in repopulated scaffolds identified the cell-matrix interactions as essential in the progression of IPF, emphasizing the BM and the underlying composition of ECM proteins as a possible disease mechanism in the induction of normal versus fibrotic tissue remodeling. We demonstrated that the IPF scaffolds had an enhanced content of proteoglycans and after the repopulation with healthy fibroblasts, we also distinguished a shift in the synthesis of proteoglycans, accompanied with a distinct localization of the tissue deposition. Together, these results further support that the existing cellular milieu alters fibroblast activity, promoting a profibrotic phenotype when cultured in a diseased matrix. More in-depth examinations with a larger number of patients on how specific ECM components may direct cellular activity are warranted to further elucidate how healthy cells become programed to synthesize a disease-like protein profile in a diseased ECM environment, studies which may lead to the unveiling of potential targets and biomarkers for IPF.

## 4. Materials and Methods

### 4.1. Decellularization of Lung Tissue Samples

Tissue samples for healthy controls originated from healthy human donor lungs, unusable for transplantation, or from an unaffected area in resection material (Table 1). The diagnosis of IPF was confirmed by clinicians with histological examination of explanted lungs that had reached end stage of disease, consistent with ERS and ATS criteria. Sample HL 4 showed no signs of pathological changes in the parenchymal tissue used for scaffold extraction.

Lung tissue was dissected for scaffold isolation within the first 24 h of surgical removal of explanted lungs. Adjacent to the pleura, cubic blocks (1 cm^3^) of parenchymal tissue were dissected from peripheral regions of the lung (Figure 1A). Tissue blocks were immediately frozen in 2-methylbutane chilled with liquid nitrogen. After storage in −80 °C lung tissue was cryosectioned into 350 µm tissue slices with a HM-560 cryostat (Microm, Heidelberg, Germany). Antifreeze cryoprotective solution (30% v/v glycerol and 30% v/v ethylene glycol in 0.1 M sodium phosphate buffer) was used to maintain the integrity of the tissue at sectioning. Tissue slices were thawed on chilled D-PBS (Dublecco’s phosphated buffer solution) (Invitrogen, Waltham, MA, USA) and treated for decellularization. Samples for histology were fixated and prepared as described below.

Tissue slices were decellularized according to Rosmark et al. [13]. In short, tissue was incubated with mild agitation in decellularization solution (8 mM CHAPS, 1 M NaCl, 25 mM EDTA in D-PBS) (1 mL/slice), with the solution replaced five times during the first 4 h of incubation. Tissue was stored overnight at +4 °C in D-PBS. The following day, tissue slices were rinsed with benzonase working buffer (20 mM Tris-HCl, 2 mM Mg^2+^, 20 mM NaCl) prior to incubation with benzonase nuclease (Sigma-Aldrich, Saint Louis, MO, USA, cat. no. E1014) (90 U/mL, 1 mL/slice, 30 min at 37 °C). The decellularized tissue (scaffold) was rinsed and stored in D-PBS supplemented with amphotericin B (2.5 µg/mL), penicillin-streptomycin (1%) and gentamicin (50 µg/mL) at +4 °C. Randomly selected scaffolds from each tissue cube were used, which further introduced biological variability between the technical replicates.

### 4.2. Study Approval

Healthy donor lung tissue explants from Lund University hospital (Lund, Sweden) and lung samples from patients diagnosed with IPF were received from Sahlgrenska University hospital (Gothenburg, Sweden). The study was approved by ethical committees in Lund and Gothenburg, Sweden (Dnr. 413/2008, 2015-891, and 1026-15). Written informed consent was received from participants or the closest relative.

### 4.3. DNA Measurements

Decellularized lung tissue was quantified for residual double stranded DNA (dsDNA) using fluorescent nucleic acid staining (Thermo Fisher Scientific, P7589). Lung tissue slices were freeze dried and homogenized with 0.1 mm zirconia silica beads (Thermo Fisher Scientific, Waltham, MA, USA, cat.no. 3488) in a fast prep bead beater (MP fastprep96, Nordic Biolabs, Täby, Sweden). Samples were centrifuged at 6000× *g* for 3 min and supernatants were analyzed for dsDNA quantification according to manufacturer’s instructions.

### 4.4. Tissue Density Measurements

For each individual within a group (i.e., biological replicates per group, *n* = 4), three technical replicates of scaffold samples were used. Scaffolds were first separately photographed to measure tissue area, using ImageJ software version 1.51n (NIH, Bethesda, MD, USA; http://imagej.nih.gov/ij) and subsequently freeze dried and weighed to calculate tissue density (mg/mm^3^).

### 4.5. Mechanical Testing

Native tissue slices and decellularized scaffolds of equal size from healthy and IPF donor tissues (*n* = 4 biological replicates per group) were mounted in organ baths (emkaBATH4, emka Technologies, Paris, France) in D-PBS for mechanical testing. Tissue samples were mounted to triangular hooks with silk suture and original tissue length was measured (L_0_). Tissues/scaffolds were then pre-loaded in tension with 350 mg by vertical elongation. After relaxation, the samples were loaded with a displacement corresponding to a strain of 5%, 10%, and 15% at a rate of 0.1 mm/s, and allowed to relax for 20 min between each sequential load step (sequential stress-relaxation). Finally, samples were tested in tension at a rate of 0.1 mm/s until they ruptured or reached a maximal additional 10 mm. The software iox 2.10.0.40 datanalystv2.6.1.18 was used for data acquisition. Tissue stiffness (N/m) was calculated from the linear region of the force displacement curve during the final tensile test, $k=\frac{F}{\delta }$, where F is the load [mN] and δ is the displacement. Ultimate force was the maximum load [mN] at failure i.e., physical breakage of the tissue.

### 4.6. Repopulation of Scaffolds with Primary Lung Fibroblasts Labeled with Heavy Arginine and Lysine

Human primary parenchymal lung fibroblasts were isolated from one healthy donor control lung as previously described [50]. Fibroblasts were expanded on regular culture flasks (Sarstedt, Nümbrecht, Germany, cat.no. 83.3910.002) in DMEM supplemented with amphotericin B (2.5 µg/mL), penicillin-streptomycin (1%), gentamicin (50 µg/mL), glutamine (1%), and 10% fetal clone serum (FCIII, Thermo Scientific) at 37 °C, 10% CO_2_. Cells in passage 7 were trypsinized and resuspended in complete SILAC DMEM Flex Media (Life Technologies, Carlsbad, CA, USA, cat.no. A2493901) supplemented with 10% dialyzed serum (Gibco, A3382001), glucose (4500 µg/mL), amphotericin B (2.5 µg/mL), penicillin-streptomycin (1%), gentamicin (50 µg/mL), 1% Glutamax along with “heavy” ^13^C_6_ labeled l-Arginine-HCl (Thermo Fisher Scientific, 88210) and “heavy” ^13^C_6_^15^N_2_-labeled l-lysine-2HCl (Thermo Fischer Scientific, 88209), as needed for optimal cell culture conditions. Fibroblasts were pre-cultured for 5 days on regular culture flasks in complete SILAC DMEM Media and scaffolds were pre-conditioned for 1 h with SILAC DMEM Media prior to re-population of scaffolds. In 24-well suspension culture plates (Sarstedt, cat.no. 83.3922.500) fibroblasts were seeded on scaffolds with mild agitation for 24 h at 10% CO_2_, 37 °C. Culture media was analyzed for cellular content for the examination of cellular attachment to scaffolds. The cell seeded scaffolds were then mounted on scaffold holders (8 mm inner diameter), composed of polyoxymethylene and incubated for up to 9 days, based on previous data [13]. Culture medium was changed after 24 h, 3 days, and 6 days of incubation. Schematics of the experimental layout is provided in Figure 1A. Repopulated scaffolds were analyzed for cellular viability after 1, 3, and 9 days of incubation (biological replicates per group *n* = 4 with two technical replicates). Cell viability was analyzed with WST-1 (Roche, Basel, Switzerland) according to manufacturer’s instructions. In brief, scaffolds were incubated at 37 °C at 10% CO_2_ with WST-1 solution (diluted in 1:10 in cell culture medium). Color development in cell medium, corresponding to cellular metabolism, was measured at 450 nm.

### 4.7. LC-MS/MS Analysis

The extraction of proteins from decellularized and repopulated lung tissue scaffolds was modified after Rosmark et al. [13]. Instead of consecutive protein extraction we here performed one protein extraction procedure. The spanned tissue area of the scaffolds (decellularized or repopulated with heavy labeled cells) were lyophilized, diluted in extraction buffer with 100 mM ammonium bicarbonate with 8 M urea, and homogenized using a Bioruptor® Plus (Diagenode SA, Seraing, Belgium) at 4 °C for 20 cycles 15 s ON/OFF. Samples were reduced with 5 mM TCEP (tris-2-carboxyethyl phosphine) 30 min at 37 °C at 850 rpm, alkylated with 10 mM IAA (iodoacetamide) for 45 min at room temperature, followed by overnight trypsin digestion at 37 °C at 300 rpm. Decellularized scaffolds samples were desalted with C18 reversed-phase spin columns (Harvard Apparatus, Holliston, MA, USA) according to manufacturer’s instructions, whereas repopulated scaffolds were desalted using SOLAμ™-SPE plates (Thermo Fisher Scientific) according to manufacturer’s instructions.

After desalting, samples were resuspended in 2% acetonitrile, 0.1% formic acid and the peptide concentrations were measured using Pierce™ Quantitative Colorimetric Peptide Assay (Thermo Scientific, Rockford, IL, USA). For all samples we adjusted the volume to inject 1 μg peptides. Peptide separations and data acquisitions were performed as previously described [13]. Briefly samples were separated on a 25 cm EASY-spray column using an EASY-nLC 1000 LC-system (Thermo Fischer Scientific) using a gradient of 5%–30% buffer B over 60 min and 30%–95% buffer B for 5 min with a flow rate of 300 nL/min. Data were acquired with a Q Exactive Plus mass spectrometer (Thermo Fischer Scientific) using top-15 data dependent acquisition (DDA) where each full mass scan covered 400–1600 *m/z* at resolution 70,000 at 200 *m/z* for both MS and MS/MS scans. MS precursor values above 1.7 × 10^4^ were required for triggering MS/MS scans. An automatic gain control (AGC) of 1 × 10^6^ with ion accumulation time of 100 ms for MS scans and 60 ms for MS/MS was used.

#### Data Analysis

MaxQuant (version 1.5.3.30) was used for analysis of raw files. Searches were performed towards a reviewed UniProt human database with standard contaminants (downloaded 2015-11-17) in Andromeda. Enzyme specificity were set for trypsin with max two missed cleavages and a mass accuracy of 4.5 ppm for precursors and 20 ppm for fragment ions. Carbamidometylation was set as fixed modification and methionine oxidation as variable. For both proteins and peptides, a false discovery rate of 1% was used. The proteomics data was deposited to the ProteomeXchange Consortium via the PRIDE partner repository [51] with the dataset identifier (PXD012322). Adjusted intensity values were calculated by multiplying raw intensity with tissue density (µg/mm^3^) specific for each patient to obtain intensity per mm^3^. For repopulated scaffolds, the dry weight of cells is assumed to be negligible and adjusted with the same density as for the original scaffold.

### 4.8. Imaging

#### 4.8.1. Scanning Electron Microscopy (SEM)

Scaffolds and native lung tissues were washed in Sorensen’s phosphate buffer (0.1 M, pH 7.4) and fixed in 1.5% formaldehyde and 1.5% glutaraldehyde for 1 h. After washing with Sorensen’s buffer, the samples were dehydrated with gradual increasing concentration of ethanol. Samples were critical point dried and sputtered with gold-palladium before being examined with electron microscopy Jeol JSM-7800F FEG-SEM at Lund University Bioimaging Center (LBIC).

#### 4.8.2. Confocal Imaging

Cells were pre-stained with Cytopainter prior to cell seeding for 3D visualization of cell distribution. Cells were trypsinized, counted, and stained using CytoPainter Cell Proliferation Stain Deep Red (Abcam, ab176736) following manufacturer’s protocol. Scaffolds were seeded as above and imaged after 1 day of culture using a Nikon Confocal A1 + microscope at LBIC. The scaffold was imaged by taking advantage of the autofluorescence, using 488 nm excitation.

#### 4.8.3. Fixation, Paraffin Embedding, and Sectioning

At selected time points scaffolds were rinsed in PBS and fixated in 4% formaldehyde (VWR; Radnor, PA, USA) for 1 h, and subsequently dehydrated immediately (70% ethanol 1 h, 95% ethanol 1 h, 99.5% ethanol 30 min, 1:1 ethanol:xylene 15 min, xylene 30 min) or stored in PBS at 4 °C. Two changes of paraffin incubation at 60 °C (1 h and 30 min) were followed by embedding into paraffin blocks, from which 4 µm thick sections were produced. Repopulated scaffolds were processed in their scaffold holder to ensure stretched morphology, and the center was punched out with a biopsy punch prior to embedding.

#### 4.8.4. Hematoxylin/Eosin Staining

After deparaffinization, hematoxylin/eosin staining was performed according to manufacturer’s instructions (Mayer’s hematoxylin, Histolab, Gothenburg, Sweden).

#### 4.8.5. Immunohistochemistry (IHC), Immunofluorescence (IF)

After deparaffinization, heat-induced epitope retrieval was performed on a PT Tissue Link system (Histolab). IHC for collagen type IV, periostin, and decorin was performed using the EnVision Dual Link System (K4065, Dako, Glostrup, Denmark) according to manufacturer’s instructions, including horse-radish peroxidase (HRP)-coupled secondary antibodies and counterstaining with Mayer’s hematoxylin to visualize nuclei. IF for collagen type VI, biglycan, versican, vimentin, and vinculin was performed by incubation with primary antibodies for 1 h and with fluorochrome-coupled secondary antibodies (Thermo Fisher Scientific, 1:200) for 45 min. Sections were mounted with ProLong Gold Antifade Mountant with DAPI (Invitrogen) to visualize nuclei. Unspecific staining of the secondary antibodies was assessed by omitting the primary antibodies (negative control). For antigen retrieval and antibody-specifications see Table 2.

#### 4.8.6. Image Acquisition

Images were obtained on a Zeiss fluorescence microscope (Nikon 4X NA 0.10 air, Nikon 10X NA 0.45 air) or with a VS120 virtual microscopy slide scanning system (Olympus, Tokyo, Japan, objectives Olympus 4X NA 0.16 air, Olympus 10X 0.4 air), either in brightfield mode (hematoxylin/eosin, IHC) or fluorescent mode (IF). From the scanned slides, representative images were acquired using the OlyVIA software 2.8 (Olympus). Exposure times, acquisition settings, and image brightness adjustments were done consistently for each respective staining including negative controls.

### 4.9. Statistics

Tissue stiffness and density were statistically analyzed with an unpaired *t*-test using software GraphPad Prism 7 (La Jolla, CA, USA). MS data were manually curated prior to statistical testing to remove single peptide hits, proteins with missing values for more than 25% per sample group for decellularized scaffolds, and proteins with 50% missing values for the repopulated samples groups.

RStudio version 1.1.442 (RStudio Team (2015). RStudio: Integrated Development for R. RStudio, Inc., Boston, MA, USA) was used for statistical analyses and for generation of heat maps, scatter plots, bar graphs, correlograms etc. Unsupervised hierarchical clustering was performed using the ward.D2 method and Euclidean distance for both row and column clusters. MS data were statistically analyzed with a Student’s *t*-test and was followed by Benjamini–Hochberg correction for multiple testing of *p*-values. Statistical evaluation of single proteins was statistically analyzed with a Student’s *t*-test treating each technical replicate as an individual data point.

## 5. Conclusions

We demonstrate how matrisome changes affect fibroblast activity using novel approaches to study temporal differences, where IPF scaffolds support a disorganized BM and upregulation of disease-associated proteins. These matrix-directed cellular responses emphasize the IPF matrisome and specifically the BM components as important factors for disease progression.

## Figures and Tables

**Table 1:** Patient and donor tissue information.

Sample ID	HL 1	HL 2	HL 3	HL 4	IPF 1	IPF 2	IPF 3	IPF 4
Group	Healthy	Healthy	Healthy	Healthy	IPF	IPF	IPF	IPF
Age	55	41	62	86	61	57	62	68
Gender	Female	Female	Male	Male	Female	Female	Female	Male
Smoking history	Yes	No	No	No	Yes	Yes	Yes	Yes
Non-lung disease				Alpha 1 anti-trypsin deficiency				
Lung disease	No	No	No	Squamous cell carcinoma *	IPF	IPF	IPF	IPF

* No COPD diagnosis. Tissue obtained from non-affected area.

**Table 2:** Antibody specifications.

Antibody	Protein Group	Catalogue Number	Dilution	HIER	Method	Secondary Antibody
Collagen type IV (α1/α2)	Collagens	Abcam, ab6586	1:4000	low pH	IHC	HRP-coupled
Collagen type VI (α1)		Abcam, ab6588	1:1000	low pH	IF	A-21246
Decorin	Proteoglycans	Atlas Antibodies, HPA003315	1:1000	high pH	IHC	HRP-coupled
Biglycan		Atlas Antibodies, HPA003157	1:500	high pH	IF	A-21246
Versican		Atlas Antibodies, HPA004726	1:500	high pH	IF	A-21246
Periostin	Glycoprotein	Abcam, ab79946	1:1000	low pH	IHC	HRP-coupled
Vimentin	Cytoskeleton	R&D, AF2105	1:200	low pH	IF	A-21432
Vinculin	Focal adhesions	Sigma-Aldrich, HPA063777	1:100	low pH	IF	A-21246

HIER = heat induced epitope retrieval; HRP = horse-radish peroxidase; A = Alexa Fluor.

## Supplementary Materials

Supplementary materials can be found at https://www.mdpi.com/1422-0067/20/16/4013/s1.
