# Supplementary material for: Matrisome Properties of Scaffolds Direct Fibroblasts in Idiopathic Pulmonary Fibrosis
Source: Int J Mol Sci. 2019 Aug 17;20(16):4013. doi: 10.3390/ijms20164013 (PMC6719040; doi:10.3390/ijms20164013)
Supplement: Supplementary file 1 [file ijms-20-04013-s001.pptx]

## Slide 1
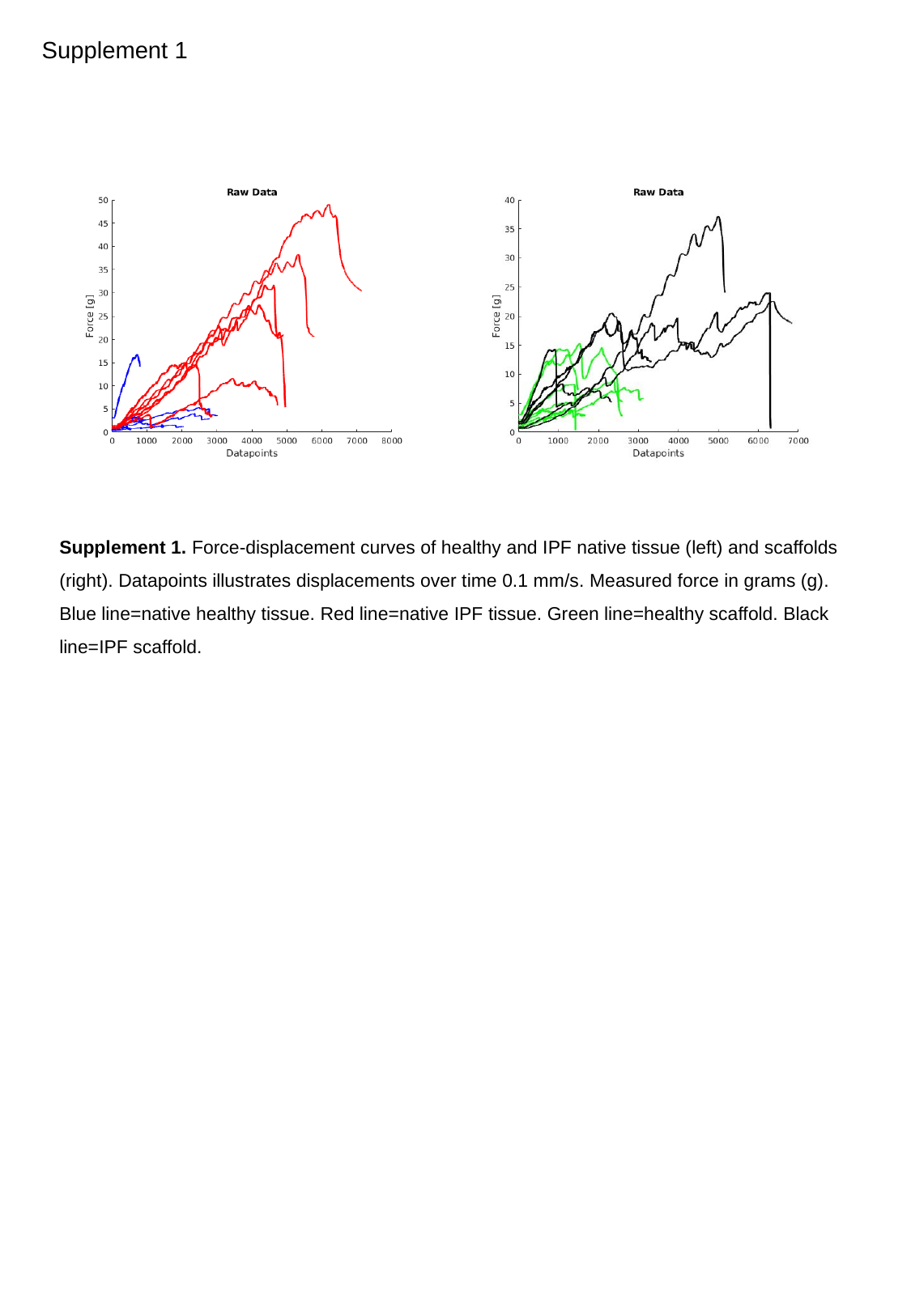

Supplement 1
Supplement 1. Force-displacement curves of healthy and IPF native tissue (left) and scaffolds (right). Datapoints illustrates displacements over time 0.1 mm/s. Measured force in grams (g). Blue line=native healthy tissue. Red line=native IPF tissue. Green line=healthy scaffold. Black line=IPF scaffold.

## Slide 2
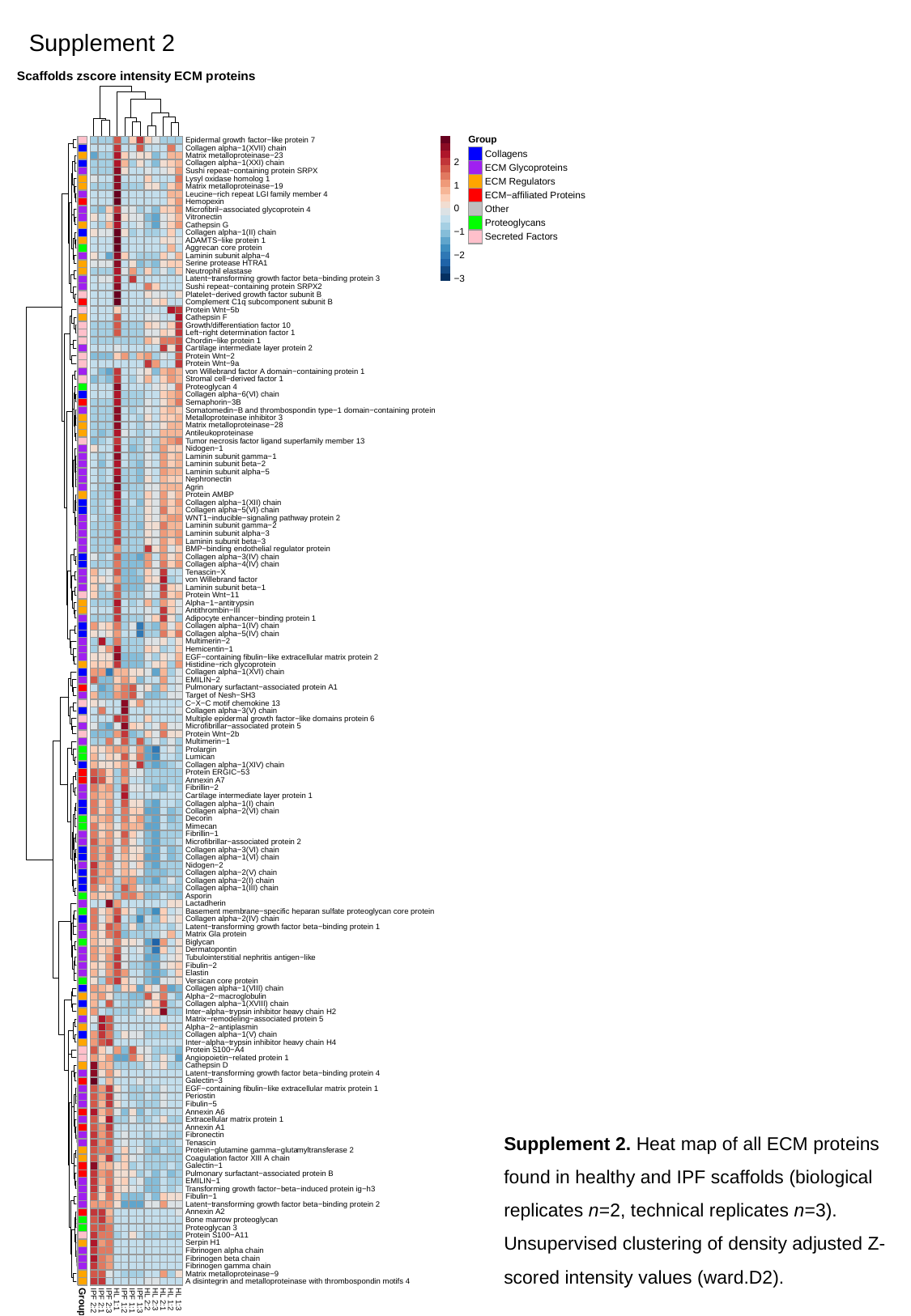

# Supplement 2
Supplement 2. Heat map of all ECM proteins found in healthy and IPF scaffolds (biological replicates n=2, technical replicates n=3). Unsupervised clustering of density adjusted Z-scored intensity values (ward.D2).

## Slide 3
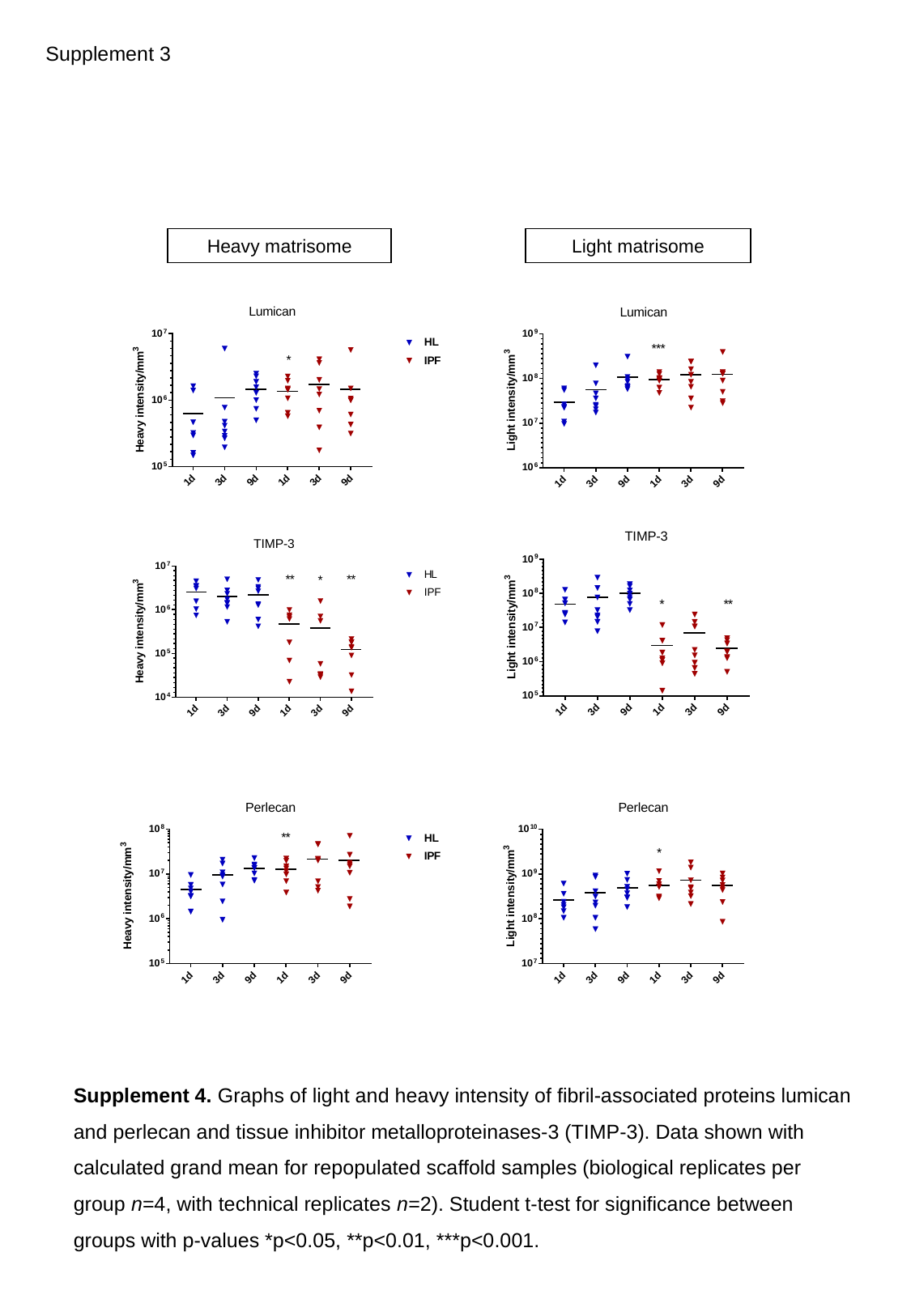

# Supplement 3
Heavy matrisome
Light matrisome
Supplement 4. Graphs of light and heavy intensity of fibril-associated proteins lumican and perlecan and tissue inhibitor metalloproteinases-3 (TIMP-3). Data shown with calculated grand mean for repopulated scaffold samples (biological replicates per group n=4, with technical replicates n=2). Student t-test for significance between groups with p-values *p<0.05, **p<0.01, ***p<0.001.

## Slide 4
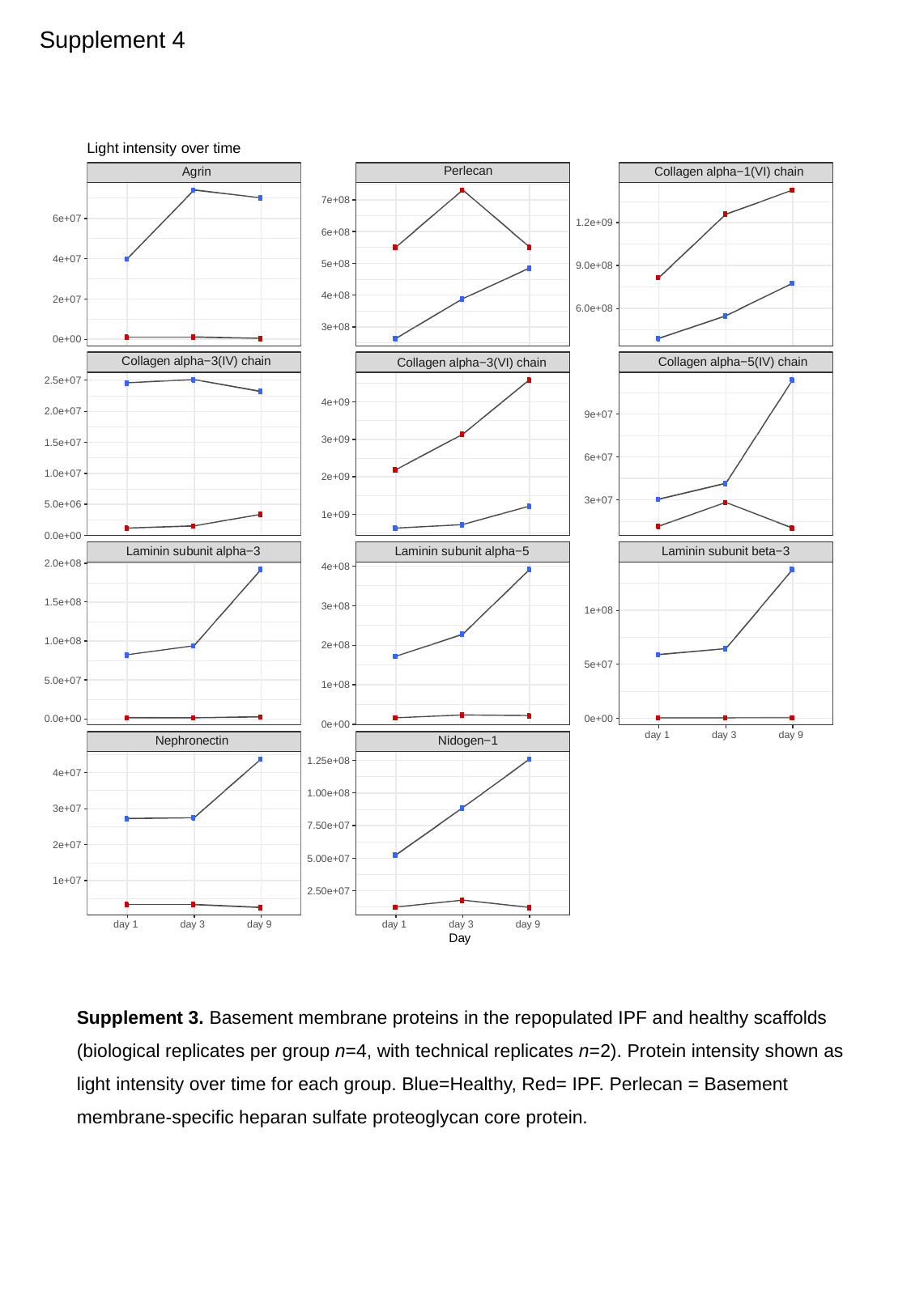

# Supplement 4
Supplement 3. Basement membrane proteins in the repopulated IPF and healthy scaffolds (biological replicates per group n=4, with technical replicates n=2). Protein intensity shown as light intensity over time for each group. Blue=Healthy, Red= IPF. Perlecan = Basement membrane-specific heparan sulfate proteoglycan core protein.

## Slide 5
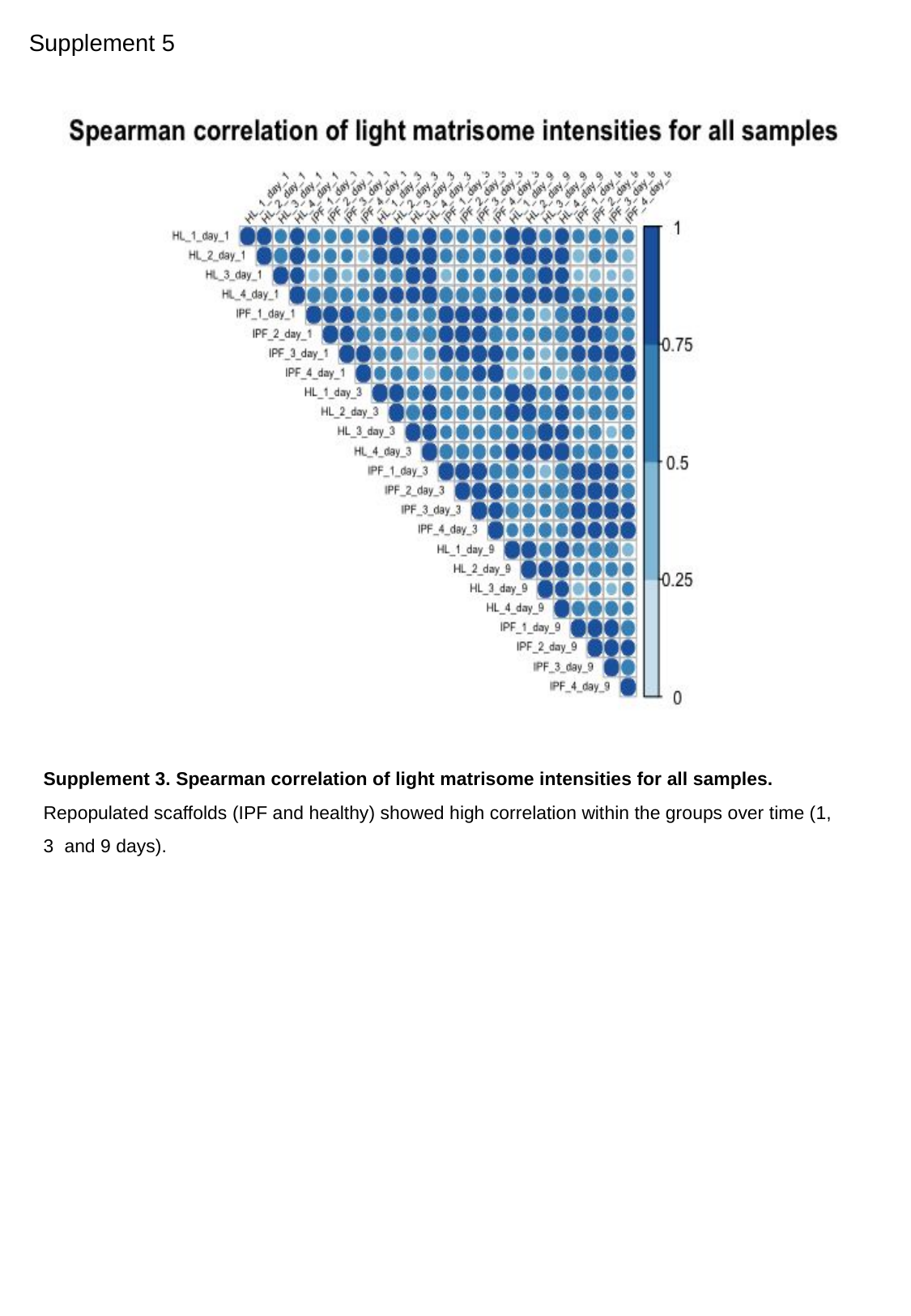

# Supplement 5
Supplement 3. Spearman correlation of light matrisome intensities for all samples. Repopulated scaffolds (IPF and healthy) showed high correlation within the groups over time (1, 3 and 9 days).
